# First plant cell atlas workshop report

**DOI:** 10.1002/pld3.271

**Published:** 2020-10-15

**Authors:** Selena Rice, Emily Fryer, Suryatapa Ghosh Jha, Andrey Malkovskiy, Heather Meyer, Jason Thomas, Renee Weizbauer, Kangmei Zhao, Kenneth Birnbaum, David Ehrhardt, Zhiyong Wang, Seung Y. Rhee, The Plant Cell Atlas Consortium

**Affiliations:** ^1^ Department of Plant Biology Carnegie Institution for Science Stanford CA USA; ^2^ Department of Biology New York University New York NY USA

**Keywords:** data science, live imaging, nanotechnology, plant cell atlas, proteomics, single‐cell sequencing

## Abstract

The societal challenges posed by a growing human population and climate change necessitate technical advances in plant science. Plant research makes vital contributions to society by advancing technologies that improve agricultural food production, biological energy capture and conversion, and human health. However, the plant biology community lacks a comprehensive understanding of molecular machinery, including their locations within cells, distributions and variations among different cell types, and real‐time dynamics. Fortunately, rapid advances in molecular methods, imaging, proteomics, and metabolomics made in the last decade afford unprecedented opportunities to develop a molecular‐level map of plant cells with high temporal and spatial resolution. The Plant Cell Atlas (PCA) initiative aims to generate a resource that will provide fresh insight into poorly understood aspects of plant cell structure and organization and enable the discovery of new cellular compartments and features. The PCA will be a community resource (www.plantcellatlas.org/) that describes the state of various plant cell types and integrates high‐resolution spatio‐temporal information of nucleic acids, proteins, and metabolites within plant cells. This first PCA initiative workshop convened scientists passionate about developing a comprehensive PCA to brainstorm about the state of the field, recent advances, the development of tools, and the future directions of this initiative. The workshop featured invited talks to share initial data, along with broader ideas for the PCA. Additionally, breakout sessions were organized around topics including the conceptual goals of the PCA, technical challenges, and community wants and needs. These activities connected scientists with diverse expertise and sparked important discussions about how to leverage and extend leading‐edge technologies and develop new techniques. A major outcome of the workshop was that the community wishes to redefine concepts of plant cell types and tissues quantitatively. A long‐term goal is to delineate all molecules within the cell at high spatio‐temporal resolution, obtain information about interacting molecular networks, and identify the contribution of these networks to development of the organism as a whole. As a first step, we wish to create comprehensive cellular and subcellular biomolecular maps of transcripts, proteins, and metabolites, track the dynamic interactions of these molecules intra‐ and intercellularly, discern complete states and transitions of specialized cell types, and integrate these disparate data points to generate testable models of cellular function. Ultimately, the PCA initiative will have a substantial positive impact by empowering a broad, diverse group of scientists to forge exciting paths in the field of plant science, facilitating connections with interested stakeholders beyond the scientific community, and enabling new agricultural technologies for a sustainable future.

## 1 INTRODUCTION

**FIGURE 1:**
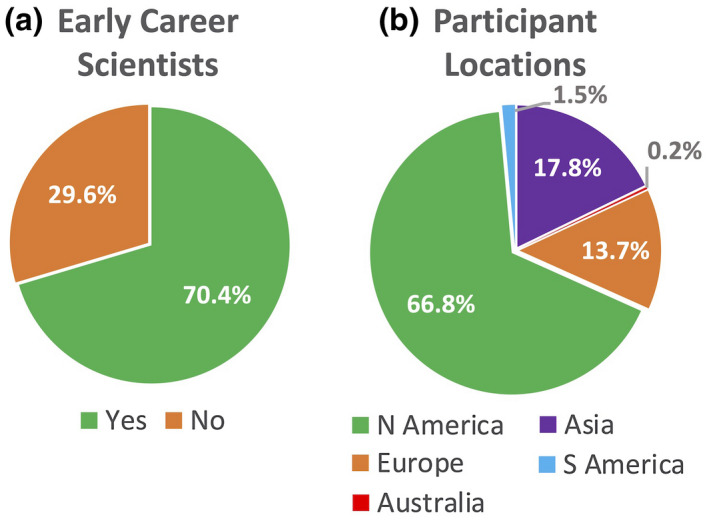
First PCA Workshop participant demographics. (a) Percentage of participants who self‐identified as early career scientists (e.g., undergraduate students, graduate students, post docs, assistant faculty, etc.). (b) Global distribution of workshop participants

The first workshop of the PCA initiative was implemented as three virtual sessions, held on May 15, May 22, and June 2, 2020 (see Appendix S1 for the workshop agenda). The goals for the sessions were to initiate the formation of a research community around the shared vision of creating a PCA by bringing together: (a) scientists addressing key biological questions in the fields of cell biology, evolutionary and developmental biology, physiology, and systems biology; (b) scientists developing new technologies in proteomics, single‐cell profiling, imaging, nanotechnology, and data analysis; and (c) scientists working in applied fields such as agriculture and bioengineering, where these technologies and knowledge will be used. Across the three sessions, 428 participants contributed their unique expertise and perspectives (see Appendix S2 for full list of participants). Notably, the participants were from diverse communities, with 70% representing scientists in early career stages (graduate students, postdoctoral fellows, or assistant faculty) and coming from 29 different countries worldwide (Figure 1).

We envisaged this first workshop as a springboard for fostering collaborations and expanding this initiative through the participants. This workshop also enabled interactions with other programs that have similarly aligned goals, such as the Human Cell Atlas project. Our international and diverse steering committee helped to ensure that our efforts were extended globally. The primary objectives of this workshop were to:

1. Create and share the vision of this initiative and build a collaborative community to develop the PCA.
2. Solicit input from the community about critical questions such as what types of information are most needed and enabling, what levels of data quality and validation are required for optimal resource utilization, and what benefits and impacts will arise for science and society.
3. Discuss data standards, data‐sharing policies, and the efforts needed to build the PCA as a scientific resource.
4. Identify and discuss relevant challenges and bottlenecks that may be encountered in this project.
5. Develop ideas and learn about emerging tools and experimental methodologies that will contribute to a high‐resolution, high‐throughput characterization of cellular proteins, including their localization, interaction, and functional attributes.

## 2 SESSION 1: VISION FOR THE PLANT CELL ATLAS

**May 15, 2020**

### 2.1 Session goals

The first session of short talks was aimed at inspiring participants to think about which biological problems could drive and benefit from the PCA initiative and how we should define the PCA as a community. The speakers shared high‐level thoughts with a diverse audience in the context of plant development/cell fate, plant–microbe interactions, organelle biogenesis, and plant/organelle engineering. The short talks were followed by a moderated Q&A session, breakout group discussions, and regrouping/summarizing. Talks and the Q&A session are available to view on the PCA website (www.plantcellatlas.org).

### 2.2 Summaries of the short talks

#### Plant development and cell fate determination

Speaker: Dominique Bergmann, PhD, Professor of Biology, Stanford University

Dr. Bergmann introduced the workshop and discussed how the PCA will inform development in plants. The talk centered on how plant cells are defined, how they are made, and how they respond to environmental changes. Transcriptome, epigenome, and proteasome analyses offer powerful methods to approximate a cell. An example is the stomatal lineage, which can serve as a model system for studying development and cell fate. Using markers of major regulators has accelerated our understanding of stomatal development. However, “missing” markers limit our ability to expand our understanding to other cell types. By contrast, a single‐cell atlas of the Arabidopsis leaf can provide more information about other cell types but is still reliant on markers for annotation.

Furthermore, advanced technologies now make it possible to map cell types and trajectories. For example, SMART‐seq can reveal differences in precursor cells, which were previously assumed to be a uniform cell type. This method allows for deeper single‐cell RNA sequencing (scRNA‐seq) coverage, providing a powerful means to identify major cell identity groups. Apart from transcriptional differences, the major governing point of cell differentiation is a differential epigenetic landscape (varied methylation or histone modification), which changes as the cell follows its developmental trajectories to form cell types. Deciphering the single‐cell epigenome during cellular development will allow a more comprehensive picture of cell types and their fate changes.

Key takeaway: The PCA will provide opportunities to capture the diversity of cell types in a broad sample of plants; studying plant cells will provide broad value for developmental biology due to their flexible development and potential for regeneration.

#### Plant–microbe interactions

Speaker: Uta Paszkowski, PhD, Professor of Plant Sciences, University of Cambridge

In nature, many plants live in intimate association with fungi. This talk focused on arbuscular mycorrhizal symbiosis: the widespread association between fungi and the inner cortex of terrestrial plant roots. This symbiosis is highly dynamic, only occurs on a small proportion of root cells, and is challenging to access experimentally due to its location in the inner cortex. Furthermore, as the life cycle of an arbuscule is only five days, it is difficult to capture by microscopy. The use of marker assisted live cell imaging has overcome these challenges and allowed researchers to visualize the dynamics over the course of arbuscule development. For example, Dr. Paszkowski’s work visualizing PT11 and ARK1, which both reside in the peri‐arbuscular membrane, revealed protein dynamics during different membrane stages. This study provided a fine‐tuned view of the temporal regulation and subcellular processes of these proteins.

Future work using these markers on the Plasticity Project 2.0 – an NSF‐PGRP‐funded collaboration – will enable multi‐level gene activity analysis. This project will leverage multiple new technologies, such as nuclear isolation (INTACT) and ribosome immunopurification (TRAP) technologies, to isolate chromatin, nuclear RNA, and ribosome‐associated mRNA in rice and other plants to evaluate the plant–mycorrhizal fungal interactions.

Key takeaway: Capturing transcriptional or translational activity in a fine‐tuned manner can provide a detailed understanding of a highly specialized, transient process in plants.

#### Organelle biogenesis

Speaker: Liwen Jiang, PhD, Professor of Life Sciences, The Chinese University of Hong Kong

Plant cells contain many organelles, the largest of which is the vacuole. Dr. Jiang discussed his work aimed at understanding vacuole development in Arabidopsis cells. In plant cells, vacuoles are classified into two populations: protein storage vacuoles (PSVs) and lytic vacuoles (LVs). Labeling tonoplasts with GFP revealed that there are vacuoles of different sizes within a single cell. In order to probe the biogenesis of vacuoles, a whole‐cell three‐dimensional (3D) electron tomography model made it possible to trace the formation of vacuoles. This method revealed small vacuoles containing intraluminal vesicles that were primarily derived from multivesicular bodies. Dr. Jiang’s work also has focused on vacuoles in guard mother cells and identified two distinct populations of vacuoles: those with and those without intraluminal vesicles. Many questions remain: What are the functions of vacuoles? What are the regulators of vacuole and cell size? What is the difference between vacuole fusion and vacuole fission?

Key takeaway: Whole‐cell 3D electron tomography provides a powerful means to view plant cell organelles in a dynamic 3D environment.

#### Organelle and plant engineering

Speaker: Martin Jonikas, PhD, Assistant Professor of Molecular Biology, Princeton University

Dr. Jonikas’ talk focused on his work on protein localization and protein–protein interactions using the pyrenoid of eukaryotic algae as a model. The goal of this work is to engineer pyrenoids into land plants to improve photosynthetic efficiency. Via an algal carbon‐concentrating mechanism, membrane tubules transport CO_2_ to the enzyme Rubisco. For a long time, scientists thought the pyrenoid was only composed of Rubisco and Rubisco activase. Using fluorescent‐tagged protein candidates, immunoprecipitation, and mass spectrometry, Dr. Jonikas’ team identified over 90 high‐confidence pyrenoid proteins. The project has become so successful that the research team is expanding this method to study thousands of proteins associated with the chloroplast. It has now become clear that the pyrenoid is a phase‐separated, liquid‐like organelle, and a linker protein holds Rubisco in the pyrenoid. The structure appears to stay constant under different CO_2_ levels, although the matrix can dissolve or condense. Unlike the pyrenoid of hornworts – the only land plant to have these structures – algal pyrenoids have both matrices and tubules. Ultimately, Dr. Jonikas’ goal is to engineer a pyrenoid into land plants to enhance crop yields.

Key takeaway: A spatially resolved protein interactome of the pyrenoid has opened the doors to its molecular characterization, illustrating the power of systematic characterization of protein localization and protein–protein interactions for the study of plant organelles.

**Open Questions**

- How can we define different cell types and states?
- How do cells respond to the environment?
- How do cells change during development?
- How can we move beyond transcriptomics to the cell, protein, and chromatin level?
- How can we resolve cellular elements at a subcellular resolution?
- How can we elucidate spatial patterning and dynamic processes?
- How can we integrate different analysis approaches?
- What are the similarities and differences between species and how can we compare cells across diverse plants?
- How can we identify genes that fulfill specialized functions?
- What are the overall functions and mechanisms of cell‐cell communication?
- How do plant cells integrate multiple networks to modulate function?

### 2.3 Breakout Session 1

Attendees were divided into 25 small breakout groups, each guided by a discussion leader. The breakout groups were tasked with addressing the following questions:

1. What are the biggest open questions in plant cellular organization and function, development, physiology, and metabolism today?

Summary Response: The breakout groups identified several important open questions in plant cellular organization, function, development, physiology, and metabolism. These questions centered around several topics: cell type classification; cell development; subcellular components; intracellular, cell–cell, and cell–environment communication; species variations; genetics and/or proteomics; metabolism; and data analysis and integration.

- How would a comprehensive PCA of protein function, molecular locations, dynamics, and interactions help address these questions?

Summary Response: To address these fundamental and long‐standing questions, the PCA will foster a community with a common goal of mapping plant cell components with high temporal and spatial resolution. The PCA will be an informative, easy‐to‐use reference platform for integrating existing information, ranging from gene to whole‐plant datasets at different ‘omics levels. This platform will be useful as both a hypothesis‐generating tool and a platform for collaboration among groups. Moreover, the PCA will empower researchers with limited access to advanced technologies and funding, providing access to relevant data that will enable researchers to form and test hypotheses. Thus, this platform will broaden the research community and ensure that data are utilized to a greater extent. Once the PCA is established, the information on this platform can be compared and potentially integrated with other existing Atlas platforms to address a broader range of biological questions.

3 What types of data should be included in a comprehensive PCA?

Summary Response: Numerous cell types, both known and unknown, have not yet been characterized at a molecular level. The PCA will facilitate research aiming to define the basic functions of plant cell types, as well as dynamic cell processes such as aging and maturing, circadian processes, and responses to environmental changes. Although single‐cell transcriptomics does not fully define the cell state, this approach exhibits immediate potential for generating a powerful preliminary resource for the study of plant cells. Beyond single‐cell transcriptomes, other modalities of gene regulation also guide cell identity. Thus, there is a critical need to assess other gene regulatory activities in plant cells to gain a comprehensive understanding of cell state and identity. Emerging techniques, such as stochastic optical reconstruction microscopy (STORM), photo‐induced force microscopy (PiFM), and real‐time live‐cell imaging, will be useful as well. Early work for the PCA could focus on genetically tractable model systems, such as Arabidopsis, maize, rice, and wheat. By leveraging mutant collections, studies can begin to link genes to single‐cell data. For species that are not as experimentally advanced, the PCA should prioritize models that best represent the wide diversity of plants used for agriculture.

4 How should efforts be prioritized in terms of species, cell, and data types?

Summary Response: As the PCA initiative develops, there will be a critical need to standardize data quality and analysis. Common terminology and parameters for experiments should be encouraged. Many factors, both biological and experimental, can adversely affect data interpretation. For example, in current practices for single‐cell RNA sequencing of plant cells, tissues must undergo protoplasting to generate single‐cell suspensions; however, this process is known to induce transcriptional changes in cells at the bulk level. Therefore, we must consider whether these transcriptional responses are uniform in all cell and tissue types. Furthermore, not all plant tissues and cell types are amenable to general protoplasting methods and, thus, require additional experimentation. The cell nucleus can be utilized as a proxy for the cell transcriptome, but this approach has both limitations and advantages. Finally, data integration and modeling represent an important yet challenging component for a successful PCA. Portals and tools that enable such data integration and modeling and that reward researchers for contributing to the community will be important.

### 2.4 Summary

In sum, the rich data of the PCA will enable a deeper understanding of plant cells, generate new questions, and allow for effective, accurate modeling. Ultimately, this resource will provide insight into crop improvements, methods for finely tuning the productivity of plants in varied environments, and targets for developing optimally engineered plants.

## 3 SESSION 2: TOOLS AND TECHNOLOGIES FOR DEVELOPING THE PLANT CELL ATLAS

**May 22, 2020**

### 3.1 Session goals

The second session of this workshop focused on recent technological advances in molecular biology, proteomics, imaging, single‐cell profiling, data science, and nanotechnology relevant for the PCA. Discussion centered on powerful tools developed in recent years, how these tools can be used broadly, and identifying new tools that can help achieve the vision of PCA. The short talks were aimed at inspiring participants to think about emerging and game‐changing technologies that are available or needed for the PCA initiative. The short talks were followed by a moderated Q&A session, breakout group discussions, and regrouping/summarizing. Talks and the Q&A session are available to view on the PCA website (www.plantcellatlas.org).

### 3.2 Summaries of the short talks

#### Emerging live imaging techniques and tools

Speaker: Alexander Jones, PhD, Research Group Leader, University of Cambridge

The first talk in this session focused on technologies for high‐resolution sensing and perturbation of plant hormones *in vivo*. Dr. Jones generated a FRET biosensor for gibberellin (GA), called the gibberellin perception sensor (GPS). This tool enables the measurement of GA at the single‐cell level, and has uncovered an intriguing correlation between GA and cell growth. Furthermore, combining optogenetic tools to manipulate gene expression in a targeted manner with the GPS sensor provides a powerful opportunity to address many unanswered questions about the biosynthesis and turnover of GA, as well as patterns and dynamics at transcriptional, translational, and regulatory levels. Future advancements could adapt this technology to study other tissues or species.

Key takeaway: Continued development of fluorescent proteins, genetically encoded sensors, and live‐cell imaging techniques can help elucidate the signaling and metabolic networks in plant cells.

#### Emerging proteomics techniques and tools

Speaker: Tess Branon, PhD, Postdoctoral Fellow, University of California, Berkeley

Cell biologists currently have a limited understanding of the complex interactions between proteins. Although transient interactions are often the most important, they are difficult to identify. Enzyme‐catalyzed proximity ligation is a promising approach for probing the spatial and interaction characteristics of proteins in living cells. However, many of the current methods have limitations, such as requiring extended labeling time or utilizing toxic chemicals. A new approach of using TurboID or miniTurboID – mutants of biotin ligase – enables fast, non‐toxic, and robust *in vivo* labeling in plants. With these tools, it is possible to capture transient interactions and perform interactome mapping. Proximity labeling does not depend on the strength of the protein–protein interaction, and can also detect interactions that are not direct, making it complementary to affinity purification mass spectrometry. TurboID requires a deprotonated lysine for the reaction to work, making it less efficient at lower pH.

Key takeaway: Recent advances in proximity labeling make it possible to define specific protein interactions and spatially or temporally restricted proteomes.

#### Emerging plant transformation techniques and tools

Speaker: Markita Landry, PhD, Assistant Professor of Chemical and Biomolecular Engineering, University of California, Berkeley

In the face of climate change and a growing world population, there is an expanding need for precise and rapid crop improvement via novel approaches. Current transformation technologies like agrobacterium‐mediated and biolistic particle delivery have several shortcomings, including the inability to control the location and frequency of transgene integration. Furthermore, genetic modification of crops is under strict regulation and requires high research and development costs. Thus, we need a plant transformation method with nanomaterial delivery that avoids these hurdles. Inorganic nanomaterials offer a means to deliver transgenes and other payloads into plants. One approach is the use of carbon nanotubes, which can transform plants in a very efficient manner, resulting in high protein expression levels without transgene integration. The nanotube protects the DNA from being degraded by the cell and prevents it from being inserted into the plant’s genome. Carbon nanotubes are designed for bench approaches rather than the field, and do not degrade over the course of study.

Key takeaway: The novel technology of carbon nanotubes offers great potential for quick and efficient gene delivery in plants across species.

#### Emerging homologous recombination techniques and tools

Speaker: Becky Bart, PhD, Associate Member, Donald Danforth Plant Science Center

This talk focused on advanced methods for tagging genes in the native genomic context in non‐model systems. Transformation takes a long time and requires a robust pipeline, and template‐directed repair is rare. Dr. Bart developed a tool for visualizing cassava bacterial blight infection by tagging MeSWEET10a, a sugar transporter that is normally expressed in flowers but is also expressed by pathogens during an infection, with GFP in its native context in cassava. Another novel tool, the SureFire Strategy, enables screening for cells with rare repair events by using a pathogen inducer. SureFire is advantageous because it identifies successful events very early in the process using a visual marker. Future work will use programmable transcriptional activators in customized settings.

Key takeaway: Novel tools allow more feasible homology‐directed repair‐based approaches for plant research.

### 3.3 Breakout Session 2

Attendees were divided into 17 breakout groups, each guided by a discussion leader. The breakout groups were tasked with addressing the following questions:

1. What are the key existing technologies and tools needed to enable a comprehensive PCA?

Summary Response: Existing methods, such as single‐cell transcriptomics, imaging, and proximity labeling, along with corresponding data‐processing tools, have been extremely useful in profiling plant behavior. Many technologies are capable of examining a narrow range of molecules (e.g., RNA, chromatin) in groups of cells, while new single‐cell methods are transformative and promising. Currently, single‐cell technologies generally are low throughput and limited to transcriptomics and epigenomics. Therefore, future work should expand single‐cell analyses to higher‐throughput platforms and expand these approaches to a more diverse array of molecule types (e.g., post‐transcriptional regulation, post‐translational modification, cell‐to‐cell dynamics, and multiple molecules within individual cells in real time).

- What technologies on the horizon (or beyond) should be developed?

Summary Response: There is a crucial need for technologies that are low cost, accessible, and reproducible across labs. Technologies on the horizon are promising, but in most cases, increasing the throughput is challenging. Moreover, methods with high throughput will need to be combined in a way that does not compromise resolution. In addition, there is an increasing need for ‘omics techniques with high spatial and temporal resolution. Currently, ‘omics techniques measure different types of molecules (e.g., DNA/RNA, proteins, metabolites, and lipids) extracted separately under denaturing conditions, and data are combined post hoc for understanding the biology. Ideally, new innovations in ‘omics that capture native‐state interactions among all types of molecules will better define biological mechanisms and pathways.

- What are the biggest technical challenges/bottlenecks and how might they be overcome?

Summary Response: Primary challenges include the application of existing approaches to tissues or plants that are recalcitrant to standard methods (e.g., opaque tissues, hard‐to‐isolate protoplasts, polyploid organisms). Technical challenges, such as autofluorescence in imaging, can hamper progress. In addition, our limited existing knowledge may hinder applications in non‐Arabidopsis species. Tools such as endogenous gene tagging and inducible and orthogonal systems will generate more information for these species. Marker‐driven technologies, such as TurboID and IntAct, can provide a greater depth of multi‐omics information for specific cell types and proteins in different environmental contexts. Further development of marker‐driven tools that enable the isolation of organelles in specific cell types could lead to more subcellular, cell type‐specific metabolomics information. These tools require concurrent development of improved imaging methods for live cells and for tissues thicker than Arabidopsis seedlings; moreover, there is a need for imaging methods that can assess a greater number of fluorescent proteins while avoiding gene silencing. Additionally, developments are needed in plant cell culture. By culturing specific cell types, some of the subcellular imaging goals of the PCA may be achieved.

### 3.4 Summary

Finally, while it is relatively easy to obtain huge quantities of data, it is difficult to integrate, analyze, interpret, validate, and translate this information into valuable knowledge. The PCA will require advanced computational resources for massive data analysis and integration, as many disparate data types must be integrated. Thus, advancements in data compilation and predictive analysis are needed. The PCA could develop recommendations to standardize methods for growing plants, isolating cells, performing experiments, reporting conditions, developing computational pipelines, and addressing specific types of datasets. Mature data analysis pipelines for ‘omics data that maintain orthology among species, along with efficient transformation methods and standardized experimental protocols can help increase the throughput of these approaches and the value of data integration among labs and across species. Increased funding, collaborative research, and cross‐disciplinary training with special focus on computational skills are key to addressing these issues.

**Table:** 

Existing technologies and remaining barriers		
Key existing technology	Technological gaps	Potential tools
*Single‐cell technologies*		
● Single‐cell transcriptomics and epigenomics ● Single‐cell chromatin accessibility ● Laser‐capture microdissection	● How to expand to other ‘omics technologies? ● How to improve throughput? ● How to improve spatial and temporal resolution? ● How to achieve reproducible cell isolation? ● How to expand to non‐model plants?	● Approaches without protoplasting ● Single‐cell proteomics, metabolomics, glycomics ● Multi‐omic profiling of single cells ● Standardized growth conditions and cell isolation protocols
*Marker‐driven technologies*		
● Proximity labeling and mass spectrometry ● TurboID ● IntAct	● How to expand marker technologies to target more organelles in specific cell types? ● How to improve sensitivity of mass spectrometry?	● Native mass spectrometry ● Tagging multiple proteins to develop a map of interactions and subcellular localizations ● Matrix‐assisted laser desorption/ionization
*Transgenic approaches*		
● Tissue/cell‐specific knockout lines	● Is this a rate‐limiting step? ● How to avoid GMO designation?	● RNA‐based CRISPR technologies ● 'Hit‐and‐run' genome editing ● Prime editing ● Epigenome editing ● Optogenetics at a single‐cell resolution ● Nanotechnology
*Imaging technologies*		
● High‐resolution imaging ● 3D/4D imaging ● Live imaging	● How to image thicker sections? ● How to overcome autofluorescence? ● How to assess multiple fluorescent proteins while avoiding gene silencing? ● How to reduce cost?	● Super‐resolution microscopy ● Cryo‐electron tomography ● High‐resolution infrared/spectral imaging ● Light sheet microscopy ● Nanotechnology ● Biosensors to track metabolites with high spatio‐temporal resolution
*Hormones/Sensors*		
● High‐throughput quantification and visualization of hormones and non‐transcriptomics data ● FRET‐based sensors	● How to improve sensitivity of biosensors? ● How to use biosensors deeper than the epidermis?	● Biosensors with dynamic range to observe cell–cell communication ● Sensors that measure secondary metabolites ● Non‐optical sensors ● Multiplex sensors
*Data analysis and interpretation*		
● Machine learning approaches ● Novel bioinformatic tools ● Modeling	● How to integrate disparate data types? ● How to increase computational and analytical capacity?	● Centralized data repository ● Data‐mining technologies ● *In silico* reconstruction of biological systems ● Ontologies and metadata standards and standard formats to ensure data are FAIR

## 4 SESSION 3: BROADER IMPACTS, INFRASTRUCTURE, AND COMMUNITY BUILDING

**June 2, 2020**

### 4.1 Session goals

The third session of this workshop focused on discussing how the PCA can impact science and society. In addition to expanding our knowledge on plant research, the PCA seeks to advance those findings into applied scientific fields. This session’s goal was to present ideas and perspectives on how to direct this initiative into building community resources such as databases and advanced biological platforms to accelerate plant science research as well as to integrate this knowledge into developing educational tools. The short talks were aimed at inspiring participants to discuss, during the breakout session, how we can create the PCA community and how this community can impact various fields of science and sectors of society. The short talks were followed by a moderated Q&A session, breakout group discussions, and regrouping/summarizing. Talks and the Q&A session are available to view on the PCA website (www.plantcellatlas.org).

### 4.2 Summaries of the short talks

#### Visualizing the PCA

Speaker: Nicholas Provart, PhD, Professor of Cell and Systems Biology

University of Toronto

This talk focused on potential methods to visualize the PCA. Dr. Provart’s group runs the Bio‐Analytic Resource for Plant Biology platform at http://bar.utoronto.ca, which facilitates the exploration of large datasets for hypothesis generation in plant biology. Recent work has focused on developing a zoomable user interface called ePlant that starts with variation of gene expression on a kilometer scale, and then moves to the centimeter scale for visualizing expression at the tissue‐level, the millimeter scale for cell‐specific expression, the sub‐millimeter scale for subcellular localization, and finally the micrometer and nanometer scales to visualize the chromosomal location of genes, protein interactors, and protein structure. Links for exploring phylogenetic relationships are also available. Future work will incorporate an ecosystem‐level module for visualizing and understanding how environmental changes relate to the various other levels.

Key takeaway: Advanced frameworks and tools will be useful for sharing, integrating, accessing, and exploring ‘omics data from plants.

#### Tissue modeling

Speaker: George Bassel, PhD, Professor of Life Sciences, University of Warwick

The organization of biological systems becomes increasingly complex when moving from genomic to phenotypic levels. Dr. Bassel’s team has helped develop a 3D digital tissue atlas of Arabidopsis across scales along with cell connectivity networks. These networks can be analyzed via data science to understand the shapes of cells, connections between cells, and the connectivity of tissues. Current work focuses on integrating these networks to understand how different network layers are connected by protein–DNA interactions.

Key takeaway: Working at the interface between biology and data science can clarify the molecular and cellular networks that control plant responses to the environment.

#### Crop engineering

Speaker: Amy Marshall‐Colón, PhD, Assistant Professor of Plant Biology, University of Illinois

The aim of Dr. Marshall‐Colón’s group is to apply integrative and multiscale modeling to build virtual crops (i.e., “Crops *in silico*”). Computational frameworks can facilitate whole‐plant simulations to understand gene expression, metabolic flux, protein synthesis, physiology, and canopy‐level growth. The goal of the Crops *in silico* platform is to provide a resource that is accessible to the entire plant biology community. The community effort involves a new user‐friendly graphical interface, journal, and meeting. However, the platform is limited by the availability and access to data; thus, the PCA will be useful in facilitating this effort.

Key takeaway: Computational models can potentially accelerate our mechanistic understanding of plant systems and predict their responses to the environment.

#### Synthetic biology and bio‐based economy

Speaker: Drew Endy, PhD, Associate Chair of Bioengineering, Stanford, University

Dr. Endy shared his experience with the International Genetically Engineered Machine (iGEM) program, which is a community of bioengineers that has expanded over time. This program has inspired people all over the world to come together with a shared goal of advancing synthetic biology. Taking this as an example, Dr. Endy encouraged the participants to consider the overall vision of the PCA. Having a positive narrative can enable collective action in advance of reality. He left participants with thought‐provoking questions of “what is the PCA’s dream?” and “where will the PCA take us?”

Key takeaway: It is important to consider the vision of the PCA and consider the possible outcomes in advance to shape the direction of this initiative. 

### 4.3 Breakout Session 3

Attendees participated in 16 breakout groups, each guided by a discussion leader. The breakout groups were tasked with addressing the following questions:

1. Who should be stakeholders/members of the PCA community?

**Key PCA Stakeholders**

- Members of the plant research community at all career stages (i.e., high school, undergraduate, and graduate students, postdoctoral fellows, early and late stage professors, professors at teaching‐focused institutions)
- Data scientists
- Industry members, from large corporations to individual farmers and breeders
- Public and private funding agencies
- Policy makers
- Consumers and other interested members of the public

- What should be the main purpose and goal of the PCA?

Summary Response: The major goals of the PCA are to share knowledge, encourage participation, and initiate new ideas in plant research. In addition to serving as a research tool, the PCA should help create a community and include educational and outreach activities. If successful, the PCA resource can connect the broader global community and provide a visual and analytical tool for efficient, in‐depth data analysis and integration. PCA stakeholders should include scientists involved in plant research as well as public and private funding agencies, policy makers, and interested members of the public, such as farmers, breeders, and consumers. The PCA will positively impact plant science by supporting communication among these various stakeholders.

The PCA should provide digestible information to the broader community while allowing people to contribute their knowledge. The PCA should establish standardization across methodologies, enabling comparative studies across tissues and species. In addition, the PCA can use its larger infrastructure expertise to build databases that house information for non‐model or emerging‐model species, as well as crop species. To be successful, the PCA must be modular and dynamic, having a team in place that is solely responsible for the curation and insertion of the latest data.

- How will the PCA impact plant science and society?

Summary Response: The PCA can impact plant science and society in general by providing a comprehensive platform of integrated data from different plant species and will be instrumental in translating basic plant science research to advance agriculture and bioenergy efforts. The PCA will pioneer data organization, accelerate applied research, identify gaps in knowledge, and coordinate funding efforts to fill those gaps. Ultimately, this initiative has the potential to spark broad interest in plant biology research and to enable solutions regarding food security associated with climate change and environmental degradation.

## 5 WORKSHOP ASSESSMENT

An important goal of the first PCA workshop was to give the participants agency and ownership of the future directions of the PCA. To this end, participant opinions were collected via polls taken during the workshop, and a survey was conducted after the workshop to assess the strengths and weaknesses of the workshop and to guide the development of future meetings (see Appendix S3 for detailed assessment results). Overall, the majority of those who completed the survey were satisfied with the various components of the workshop and felt that the PCA workshop was relevant and helpful to their research. Many participants commented that the workshop was successful, especially considering the last‐minute change from an in‐person setting to a virtual format and appreciated the balance between talks and breakout sessions.

Participants were also queried regarding their broad goals for the PCA. According to the poll results, participants felt that the most critical missing datasets relevant to the PCA are live imaging data of protein localization and single‐cell sequencing/omics datasets; similarly, imaging and single‐cell sequencing were reported as the most useful techniques for this community. The participants identified the biggest gaps in the field as the need for standardization in data analysis pipelines and platforms, studies correlating phenotypes with genomics datasets, and information on large‐scale protein interactions and complexes. Overall, the participants hope that the PCA will become a comprehensive and dynamic platform of integrated data from different plant species that establishes standards, collates datasets, and celebrates achievements that work towards a complete spatial and temporal understanding of plant growth and development.

Looking ahead, the community expressed a desire for future webinars and two‐hour workshops on specific topics such as single‐cell sequencing, with recordings of these meetings being available via the PCA website. Several participants commented that future workshops should allot more time for each short talk to allow for a deeper dive into cutting‐edge research and techniques in the plant field. Others requested more opportunities for networking, particularly for early career scientists, which was challenging to incorporate in a virtual format. Participants also stated that the PCA could benefit from communicating through a Gordon‐style conference, a section in a major plant international meeting, a digital meeting dedicated to the PCA, or an ongoing section in a plant journal or from liaising with the American Society of Plant Biologists or the Human Cell Atlas. The majority also reported that a PCA discussion email list would be useful. Importantly, participants felt that the PCA community should uphold values focused on transparency and the provision of open data and source codes, as a centralized, accessible resource can potentially propel the plant field forward and inspire new research directions, technologies, and collaborations.

## 6 CONCLUSIONS AND OUTLOOK

To completely understand how a plant grows and functions as a self‐organizing system, we need knowledge about the tissues, cell types, and developmental stages in which each gene is expressed, the subcellular location of each protein, protein–protein interactions in various cell types, and the distribution and transport of metabolites among cells and tissues. The overarching goal of the PCA initiative is to create a community resource that describes the state and organization of various cell types at high resolution. Major advancements in technology, such as systems biology, biosensors, and high‐resolution single‐cell ‘omics, have already enabled unprecedented discoveries in plant science. To better understand how these different levels of organization relate to one another to drive form and function, we need to establish a community and crowd‐source data, knowledge, and skills under a single shared platform.

The development of new tools and technologies, such as single‐cell ‘omics and advanced live imaging, has enabled researchers to address powerful questions in plant biology. Nevertheless, to continue achieving meaningful advances, the PCA community concluded that more technologies must be developed. For instance, there is a need for a centralized data‐sharing platform to attain more comprehensive data integration and visualization, with sophisticated artificial intelligence technologies for uncovering new trends in large datasets that are currently difficult to identify. These developments will ultimately foster and complement smarter experimental tools, such as synthetic biology and bioengineering modules, which will be critical for systematically exploring novel research areas and ideas. Connecting these elements will help us overcome many of the bottlenecks and challenges currently faced by plant researchers.

Importantly, the PCA will make plant science and molecular biology accessible to a larger audience. The PCA can empower researchers – even those with limited resources – to study important research questions. With a user‐friendly interface, the PCA can also serve as a powerful resource for the public, enhancing education and providing translational opportunities for applied scientific fields such as bioenergy, agriculture, medicine, and earth sciences.

## Supporting information

Appendix S1‐S4

