# Supplementary material for: First plant cell atlas workshop report
Source: Plant Direct. 2020 Oct 15;4(10):e00271. doi: 10.1002/pld3.271 (PMC7557347; doi:10.1002/pld3.271)
Supplement: Supplementary file 1 — Appendix S1‐S4 [file PLD3-4-e00271-s001.pdf]

Appendix 1: Workshop Agenda

Session 1: Vision for the Plant Cell Atlas

May 15, 2020

8:00 AM - 8:10 AM Introduction
8:10 AM - 8:30 AM Short Talks

- **Dominique Bergmann** (Stanford University) - *Plant development and cell fate determination*
- **Uta Paszkowski** (University of Cambridge) - *Plant-microbe interactions*
- **Liwen Jiang** (The Chinese University of Hong Kong) - *Organelle biogenesis*
- **Martin Jonikas** (Princeton University) - *Organelle and plant engineering*

8:30 AM - 8:45 AM Q&A Session
8:45 AM - 9:30 AM Breakout Session 1: Scientific challenges
9:30 AM - 10:00 AM Rejoin and Report-out

Session 2: Tools and Technologies for developing the Plant Cell Atlas

May 22, 2020

8:00 AM - 8:10 AM Introduction
8:10 AM - 8:30 AM Short Talks

- **Alexander Jones** (Sainsbury Laboratory) - *Emerging live imaging techniques & tools*
- **Tess Branon** (University of California, Berkeley) - *Emerging proteomics techniques & tools*
- **Markita Landry** (University of California, Berkeley) - *Emerging plant transformation techniques & tools*
- **Becky Bart** (Danforth Plant Science Center) - *Emerging homologous recombination techniques & tools*

8:30 AM - 8:45 AM Q&A Session
8:45 AM - 9:30 AM Breakout Session 2: Technology
9:30 AM - 10:00 AM Rejoin and Report-out

Session 3: Broader Impacts, Infrastructure and Community Building

June 2, 2020

8:00 AM - 8:10 AM Introduction
8:10 AM - 8:30 AM Short Talks

- **Nicholas Provart** (University of Toronto) - *Visualizing the PCA*
- **George Bassel** (University of Warwick) - *Tissue modeling*
- **Amy Marshall-Colón** (University of Illinois Urbana-Champaign) - *Crop engineering*
- **Drew Endy** (Stanford University) - *Synthetic biology and bio-based economy*

8:30 AM - 8:45 AM Q&A Session
8:45 AM - 9:30 AM Breakout Session 3: Broader Impacts + Community Building
9:30 AM - 9:55 AM Rejoin and Report-out
9:55 AM - 10:00 AM Closing Remarks by Carnegie President Eric D. Isaacs

Appendix 2: Workshop Participants

Organizing Committee

Sue Rhee, Carnegie Institution for Science
Selena Rice, Carnegie Institution for Science
David Ehrhardt, Carnegie Institution for Science
Kenneth Birnbaum, New York University
Zhiyong Wang, Carnegie Institution for Science
Emily Fryer, Carnegie Institution for Science
Suryatapa Ghosh Jha, Carnegie Institution for Science
Heather Meyer, Carnegie Institution for Science
Kangmei Zhao, Carnegie Institution for Science
Jason Thomas, Carnegie Institution for Science
Renee Weizbauer, Carnegie Institution for Science
Andrey Malkovskiy, Carnegie Institution for Science

Steering Committee

Jim Haseloff, Cambridge University
David Jackson, Cold Spring Harbor Laboratory
Edward Marcotte, University of Texas at Austin
John Marioni, European Bioinformatics Institute
Marisa Otegui, University of Wisconsin
Nicholas Provart, University of Toronto
Alberto Salleo, Stanford University
Waltraud Schulze, University of Hohenheim
Edgar Spalding, University of Wisconsin
Michael Sussman, University of Wisconsin
Marja Timmermans, University of Tübingen
H-S Phillip Wong, Stanford University

Breakout Group Discussion Moderators

Amir Ahkami, Pacific Northwest National Laboratory
Julia Bailey-Serres, University of California, Riverside
George Bassel, University of Warwick
Philip Benfey, Howard Hughes Medical Institute, Duke University
Elison Blancaflor, Noble Research Institute
Steven Briggs, University of California, San Diego
Cesar Cuevas-Velazquez, Facultad de Química, UNAM
Peter Denolf, BASF
Diane Dickel, Lawrence Berkeley National Lab
Dave Ehrhardt, Carnegie Institution for Science
Moises Exposito-Alonso, Carnegie Institution for Science,
Christine Faulkner, John Innes Centre
Juan Pablo Giraldo, University of California, Riverside

Maria Harrison, Boyce Thompson Institute
Carol Huang, New York University
David Jackson, Cold Spring Harbor Laboratory
Song Li, Virginia Tech
Christophe Liseron-Monfils, BASF
Tie Liu, University of Florida
Heather Meyer, Carnegie Institution for Science
Blake Meyers, Donald Danforth Plant Science Center
Lukas Mueller, Boyce Thompson Institute
Naomi Nakayama, Imperial College London
Luke Nikolov, University of California, Los Angeles
Marisa Otegui, University of Wisconsin, Madison
Anna Stepanova, North Carolina State University
Sarah Van Dierdonck, Duke University
Zhiyong Wang, Carnegie Institution for Science
Clay Wright, Virginia Tech
Tingting Xiang, University of North Carolina, Charlotte
Kangmei Zhao, Carnegie Institution for Science

Breakout Group Discussion Scribes

Amir Ahkami, Pacific Northwest National Laboratory
Ahmet Bakirbas, University of Massachusetts, Amherst
Rebecca Bart, Donald Danforth Plant Science Center
Tanya Berardini, TAIR/Phoenix Bioinformatics
Alexander Borowsky, University of California, Riverside
Tess Branon, University of California, Berkeley
Jenn Brophy, Stanford University
Chai Hao Chiu, University of Cambridge
Benjamin Cole, DOE-Joint Genome Institute
Cesar Cuevas-Velazquez, Facultad de Química, UNAM
Dave Ehrhardt, Carnegie Institution for Science
Noah Fahlgren, Donald Danforth Plant Science Center
Emily Fryer, Carnegie Institution for Science
Maria Harrison, Boyce Thompson Institute
Carol Huang, New York University
Sury Jha, Carnegie Institution for Science
Lothar Kalmbach, University of Cambridge
Elena Lazarus, Carnegie Institution for Science
Travis Lee, Salk Institute and Howard Hughes Medical Institute
Marc Libault, University of Nebraska-Lincoln
Dianyi Liu, Donald Danforth Plant Science Center and University of Missouri, St. Louis
Camila Lopez-Anido, Stanford University
Yongxian Lu, Carnegie Institution for Science
Andrey Malkovskiy, Carnegie Institution for Science
Natasha Metzler, Carnegie Institution for Science

Heather Meyer, Carnegie Institution for Science
Blake Meyers, Donald Danforth Plant Science Center
Tatsuya Nobori, Salk Institute
Nicholas Provart, University of Toronto
Leonore Reiser, TAIR/Phoenix Bioinformatics
Ellen Rim, Stanford University
Marcela Rojas-Pierce, North Carolina State University
Lacey Samuels, University of British Columbia, Vancouver
Ying Sun, University of California, Riverside
Joseph Swift, Salk Institute and Howard Hughes Medical Institute
Jason Thomas, Carnegie Institution for Science
Renee Weizbauer, Carnegie Institution for Science
Clay Wright, Virginia Tech
Kangmei Zhao, Carnegie Institution for Science

Participants

Mingkee Achom, Cornell University
Muhammad Adik, University of Karachi
Pinky Agarwal, National Institute of Plant Genome Research
Amanda Agosto Ramos, University of California, Davis
Amir Ahkami, Pacific Northwest National Laboratory
Garo Akmakjian, University of California, Riverside
Jahed Ahmed, Louvain Institute of Biomolecular Science and Technology, UCLouvain
Oluwafemi Alaba, University of Maine
Milad Alizadeh, University of British Columbia
Gazala Ameen, Washington State University
Christopher Anderton, Pacific Northwest National Laboratory
Ximena Anleu Gil, University of California, Davis
Christine Ann Aquino, Carnegie Institution for Science
Cris Argueso, Colorado State University
Jaskaran Kaur Arora, Punjab Agricultural University
Sumedha Arora, Punjab Agricultural University
Deepika Arora, Indian Institute of Technology, Fulbright Scholar Program
Mario Arteaga-Vazquez, Universidad Veracruzana
Alok Arun, Inter American University of Puerto Rico
Arif Ashraf, University of Massachusetts, Amherst
Julia Bailey-Serres, University of California, Riverside
Bhaskar Bajaru, ICAR-National Bureau of Plant Genetic Resources
Ahmet Bakirbas, University of Massachusetts, Amherst
Vimal Kumar Balasubramanian, Pacific Northwest National Laboratory
Bastiaan Bargmann, Virginia Tech
Rebecca Bart, Donald Danforth Plant Science Center
George Bassel, University of Warwick
Vijaya Batthula, Cornell University
Johanna Bautista, University of California, Davis

Philip Benfey, Howard Hughes Medical Institute, Duke University
Tanya Berardini, TAIR/Phoenix Bioinformatics
Dominique Bergmann, Stanford University
Hannah Berry, Colorado State University
Vishnu Bhat, University of Delhi
Kaushal Kumar Bhati, Louvain Institute of Biomolecular Science,UCLouvain, Belgium
Devaki Bhaya, Carnegie Institution for Science
Chao Bian, University of California, Davis
Amir J Bidhendi, McGill University
Elison Blancaflor, Noble Research Institute
Bernhard Blob, Cambridge University
Andrea Bollmann, None
Alessandra Bonfanti, Cambridge University
Michiel Bontinck, VIB Tech Watch
Navadeep Boruah, Carnegie Institution for Science
Alexander Borowsky, University of California, Riverside
Flavia Bossi, Carnegie Institution for Science
Matthieu Bourdon, Cambridge University
Tess Branon, University of California, Berkeley
Emily Breeze, University of Warwick
Steven Briggs, University of California, San Diego
Jenn Brophy, Stanford University
Javier Brumos, North Carolina State University
Tawni Bull, University of California, Davis
Tony Burdett, European Bioinformatics Institute
Danbi Byun, Carnegie Institution for Science
Victoria Calatrava, Carnegie Institution for Science
Alex Canto-Pastor, University of California, Davis
Tianjun Cao, Westlake University
Clay Carter, University of Minnesota
Keri Cavanaugh, University of California, Davis
Alan Cervantes-Pérez, Langebio-Cinvestav
Zhongyuan Chang, Cornell University
Thanin Chantarachot, University of California, Riverside
Shiyan Chen, Cornell University
Han-Yi Chen, North Carolina State University
Meng Chen, University of California, Riverside
Yuan Chen, University of California, Berkeley
Sabrina Chin, Noble Research Institute
Chai Hao Chiu, University of Cambridge
Wah Chiu, Stanford University
Steve Clouse, National Science Foundation
Josh Cohn, Syngenta
Benjamin Cole, DOE-Joint Genome Institute
Maite Colinas, ETH Zurich

Kevin Cox, Donald Danforth Plant Science Center
Alfredo Cruz, Unit of Advanced Genomics-CINVESTAV
Elli Cryan, University of California, Davis
Cesar Cuevas-Velazquez, Facultad de Química, UNAM
Cary Cui, North Carolina State University
Yong Cui, Chinese University of Hong Kong
Shuaijian Dai, Hong Kong University of Science and Technology
Abhaya Dandekar, University of California, Davis
Benoit Danilo, Michigan State University
Jaishri Rubina Das, National Institute Of Plant Genome Research
Tapos Kumar Das, Research Associate, Dept. of Architecture, BRAC University
Lisa David, University of Florida
Philip Day, Washington State University
Renata de Almeida, University of California, Davis
Juan Carlos del Pozo, Centro de Biotecnologia y Genómica de Plantas
Gozde Demirer, University of California, Berkeley
Peter Denolf, BASF
Tom Denyer, University of Tuebingen
Luigi Di Costanzo, University of Naples Federico II
Luis Díaz-Batalla, Universidad Politécnica de Francisco I. Madero
Diane Dickel, Lawrence Berkeley National Lab
Stephen DiFazio, National Science Foundation
Georgia Drakakaki, University of California, Davis
Yuanlin Duan, Carnegie Institution for Science
Joseph Ecker, Salk Institute and Howard Hughes Medical Institute
Dave Ehrhardt, Carnegie Institution for Science
Drew Endy, Stanford University
Isil Erbasol Serbes, University of Bremen
Alice Eseola, The Sainsbury Laboratory Norwich
Matt Evans, Carnegie Institution for Science
Moises Exposito-Alonso, Carnegie Institution for Science,
Noah Fahlgren, Donald Danforth Plant Science Center
Andrew Farmer, National Center for Genome Resources
Christine Faulkner, John Innes Centre
Alvaro Daniel Fernandez, VIB-UGent Center for Plant Systems Biology
Camilla Ferrari, VIB-UGent Center for Plant Systems Biology
Maria Ferrer, University of California, Davis
Dulce Flores, Universidad Veracruzana
Lorenzo Frigerio, University of Warwick
David Gang, Washington State University
Nancy George, European Bioinformatics Institute
Stefania Giacomello, SciLifeLab
Adeshpal Singh Gill, Punjab Agricultural University
Jesse Gillis, Cold Spring Harbor Laboratory
Danny Ginzburg, Carnegie Institution for Science

Juan Pablo Giraldo, University of California, Riverside
Senthilraja Givindasamy, Tamil Nadu Agricultural University
Shipra Goel, Delhi Technological University
Camila Goldy, Institute of Molecular and Cellular Biology of Rosario
Victoria Gomez, Centre National de la Recherche Scientifique
Tanya Gómez, Instituto de Biotecnología y Ecología Aplicada
Fabio Gomez-Cano, Michigan State University
Jerry González, San Carlos University of Guatemala
Eduardo Gonzalez, University of California, Berkeley
Mary Paz Gonzalez-Garcia, CBGP-UPM, Spain
Mona Gouran, University of California, Davis
Sharon Greenblum, Joint Genome Institute
Joe Grosskopf, University of California, Davis
Han Han, University of California, Riverside
Pubudu Handakumbura, Pacific Northwest National Laboratory
Maria Harrison, Boyce Thompson Institute
Jim Haseloff, Cambridge University
Elizabeth Haswell, Washington University in St. Louis
Wanying He, Julius-Kuehn Institute
Adrian Hegeman, University of Minnesota
Venura Herath, University of Peradeniya
Venura Herath, Texas A&M University
Ryan Hoy, University of British Columbia
Carol Huang, New York University
Linzhou Huang, North Carolina State University
Weichao Huang, Carnegie Institution for Science
Allen Hubbard, Danforth Plant Science Center
Matthew Huff, University of Tennessee Institute of Agriculture
Maureen Hummel Gateas, University of California, Riverside
Garret Huntress, Carnegie Institution for Science
Huan Huo, University of California, Davis
Alfred Huo, University of Florida
Natanella Illouz-Eliaz, Hebrew University in Jerusalem
Lani Irvin, Middle Georgia State University
Eric Isaacs, Carnegie Institution for Science
Zia Ul Islam, Government Post Graduate College Bannu
Alon Israeli, Hebrew University
Janet Iwasa, University of Utah
David Jackson, Cold Spring Harbor Laboratory
Mohini Jaiswal, National Institute of Plant Genome Research
Pankaj Jaiswal, Oregon State University
Pratibha Jakhu, Punjab Agricultural University, Ludhiana
Adriana Jelinkova, Institute of Experimental Botany
Sury Ghosh Jha, Carnegie Institution for Science
Yuling Jiao, Institute of Genetics and Developmental Biology

Catherine Jimenez, University of Worcester
Robert Jinkerson, University of California, Riverside
Leonardo Jo, University of California, Davis
Diane Jofuku Okamuro, National Science Foundation
Alexander Jones, Cambridge University
Martin Jonikas, Princeton University
Henrik Jönsson, University of Cambridge
Sanjay Joshi, University of Kentucky
Lydia-Marie Joubert, Stanford University
Florian Jupe, Bayer Crop Science
Lothar Kalmbach, University of Cambridge
Shrikaar Kambhampati, Donald Danforth Plant Science Center
Purva Karia, University of Toronto
Isuru Kariyawasam, Edinburgh University
Kerstin Kaufmann, Humboldt-Universitaet zu Berlin
Amandeep Kaur, Punjab Agricultural University
Navneet Kaur, Punjab Agricultural University
Simranjot Kaur, Punjab Agricultural University
Harmanpreet Kaur, Punjab Agricultural University
Dorota Kawa, University of California, Davis
Taiji Kawakatsu, National Agriculture and Food Research Organization
Caroline Keller, University of California, Davis
Imran Khan, North Carolina State University
Mather Khan, University of Missouri
Dae Kwan Ko, Michigan State University
Hasan Kolkas, Paul Sabatier University
Shuyao Kong, Cornell University
Chen Kuang, Chinese Academy of Agricultural Sciences
Pankaj Kumar, SERB-National Post Doctoral Fellowship, CSIR-IHBT Palampur Himachal Pradesh
Pradeep Kumar, Centre for Cellular and Molecular Biology
Sagar Kumar, Mata Gujri College
Sumit Kumar, Indian Institute of Maize Research
Arun Kumar, CSIR-Institute of Himalayan Bioresource Technology
Vivek Kumar, Cold Spring Harbor Laboratory
Pankaj Kumar, Institute of Plant Genetics
Sunita Kumari, Cold Spring Harbor Laboratory
Priyanka Kumari, National Institute of Plant Genome Research
Markita Landry, University of California, Berkeley
Dhruv Lavania, University of Alberta
Elena Lazarus, Carnegie Institution for Science
Jun Lee, University of Tennessee
Travis Lee, Salk Institute and Howard Hughes Medical Institute
Jiyoung Lee, Virginia Tech
Samuel Leiboff, Oregon State University

Bryan Leong, Michigan State University
Tedrick Salim Lew, Massachusetts Institute of Technology
Lingjun Li, University of Wisconsin, Madison
Ning Li, Hong Kong University of Science and Technology
Song Li, Virginia Tech
Xu Li, North Carolina State University
Rong Li, University of Hohenheim
Xiaobo Li, Westlake University
Marc Libault, University of Nebraska-Lincoln
Jer-Young Lin, Academia Sinica
Penelope Lindsay, Cold Spring Harbor Laboratory
Christophe Liseron-Monfils, BASF
Dianyi Liu, Donald Danforth Plant Science Center and University of Missouri, St. Louis
Helen Liu, University of California, Berkeley
Tie Liu, University of Florida
Zhiqi Liu, Chinese University of Hong Kong
Lorinda Loitongbam, National Institute of Plant Genome Research
Ansul Lokdarshi, University of Tennessee
Tamar Lolua, Carnegie Institute for Science
Montserrat Lopez Coria, Facultad de Química, UNAM
Jennifer López Ortiz, University of Helsinki
Camila Lopez-Anido, Stanford University
Loitongbam Lorinda Devi, National Institute of Plant Genome Research
Yongxian Lu, Carnegie Institution for Science
Leonie Luginbuehl, University of Cambridge
Xiaoli Ma, University of Tuebingen
Jaspreet Maan, Punjabi University
Iain Macaulay, Earlham Institute
Sakil Mahmud, University of Bonn
Andrea Mair, Stanford University and Howard Hughes Medical Institute
Kamal Kumar Malukani, Centre for Cellular and Molecular Biology
Concepcion Manzano, University of California, Davis
Edward Marcotte, University of Texas at Austin
John Marioni, European Bioinformatics Institute
Amy Marshall-Colon, University of Illinois Urbana-Champaign
Javier Martinez, Leloir Institute
Grace Alex Mason, University of California, Davis
Ann McElwain, Carnegie Institution for Science
Claire McWhite, University of Texas at Austin
Karina Medina-Jimenez, Arkansas Biosciences Institute
Miranda Meents, University of British Columbia
Molly Megraw, Oregon State University
Devang Mehta, University of Alberta
Nick Melosh, Stanford University
Natasha Metzler, Carnegie Institution for Science
Blake Meyers, Donald Danforth Plant Science Center

Javier A Miret, University of Reading
Shruti Mishra, National Institute of Plant Genome Research
Jenny Mortimer, Lawrence Berkeley National Laboratory
Lukas Mueller, Boyce Thompson Institute
Casey Murphy, Universiteit Ghent
Katherine Murphy, University of California, Davis
Masayoshi Nakamura, Nagoya University
Naomi Nakayama, Imperial College London
Yang Nan, Hong Kong University of Science and Technology
Pavithran Narayanan, University of Delhi
Hilde Nelissen, VIB-UGent Center for Plant Systems Biology
Sihui Ni, North Carolina State University
Luke Nikolov, University of California, Los Angeles
Ido Nir, Stanford University
Fangfang Niu, Chinese University of Hong Kong
Tatsuya Nobori, Salk Institute
Trevor Nolan, Duke University
Ronan O'Malley, Joint Genome Institute
Toshihiro Obata, University of Nebraska-Lincoln
Aaron Ogden, Pacific Northwest National Labs
Uwe Ohler, Humboldt University
Toluwase Olukayode, University of Saskatchewan
Mercy Oluokun, American Society of Plant Biologists
Marisa Otegui, University of Wisconsin, Madison
Sofia Otero, University of Cambridge
JhengYang Ou, Sinica
Takehiro Ozawa Uyeda, Cinvestav-IPN Irapuato
Thirunarayanan P., Banaras Hindu University
Kannan Pachamuthu, National Center for Biological Sciences
Gergo Palfalvi, National Institute for Basic Biology
Shirsa Palit, Indian Institute of Science Education and Research Pune
Kristen Palumbo, Carnegie Institution for Science
Xue Pan, University of California, Riverside
Dilip Panthee, North Carolina State University
Michael Passalacqua, Cold Spring Harbor Laboratory
Sunita Pathak, Donald Danforth Plant Science Center
Priyanka Paul, University of Kentucky
Puneet Paul, University of Nebraska-Lincoln
Michelle Pazmino, Carnegie Institution for Science
Yunru Peng, Carnegie Institution for Science
Zoee Perrine, Donald Danforth Plant Science Center
Olga Pontes
Montcharles Pontes, Mato Grosso do Sul State University
Elly Poretsky, University of California, San Diego
Ramgopal Prajapati, National Institute Of Plant Genome Research

Jesus Preciado, University of Florida
Matthew Prior, University of California, Riverside
Nicholas Provart, University of Toronto
Elsa Herminia Quezada, Universidad Nacional Autónoma de México
Teagen Quilichini, National Research Council Canada
Carlos Quiros, University of California, Davis
Dhanya Ramadurai, University of Madras
John Randell, Human Cell Atlas
Bikash Raul, National Institute of Plant Genome Research
Leonore Reiser, TAIR/Phoenix Bioinformatics
Mauricio Reynoso, Instituto de Biotecnología y Biología Molecular
Felix Rico, Laboratorio Nacional de Genómica para la Biodiversidad
Ellen Rim, Stanford University
Marcela Rojas-Pierce, North Carolina State University
Joel Rodriguez-Medina, University of California, Davis
Sarah Rommelfanger, Washington University in St. Louis and Donald Danforth Plant Science Center
Ronelle Roth, University of Cambridge
Rahul Roy, University of Minnesota
Yue Rui, Stanford University
Kumud Saini, National Institute of Plant Genome Research
Asif Saleem, University of Central Punjab
Alberto Salleo, Stanford University
Steven Salvini, Heriot-Watt University
Lacey Samuels, University of British Columbia, Vancouver
Lekshmy Sathee, Indian Agricultural Research Institute
Maite Saura Sanchez, Instituto de Investigaciones Fisiológicas y Ecológicas vinculadas a la Agricultura
Craig Schenck, Michigan State University
Waltraud Schulze, University of Hohenheim
Heike Sederoff, North Carolina State University
Kumar Selvaraj, University of Saskatchewan
Iris Sevilem, University of California, Davis
Carolin Seyfferth, VIB for Plant Science
Lidor Shaar-Moshe, University of California, Davis
Rachel Shahan, Duke University
Priyanka Sharma
Jinbo Shen, Zhejiang A&F University
Ruben Shrestha, Carnegie Institution for Science
Rosalie Sinclair, University of California, Davis
Dalvir Singh, Punjab Agricultural University
Manjinder Singh, Punjab Agricultural University
Rajveer Singh, Punjab Agricultural University
Prabhjot Singh
Shyam Solanki, Washington State University

Edgar Spalding, University of Wisconsin
Anjil Srivastava, Durham University
Deevita Srivastava, National Institute of Plant Genome Research
Margaret Staton, University of Tennessee, Knoxville
Anna Stepanova, North Carolina State University
Josh Strable, Cornell University
Peipei Sun, Donald Danforth Plant Science Center
Ying Sun, University of California, Riverside
Michael Sussman, University of Wisconsin
Joseph Swift, Salk Institute and Howard Hughes Medical Institute
Anne Sylvester, National Science Foundation
Isaiah Taylor, Duke University
Marcela K Tello-Ruiz, Cold Spring Harbor Laboratory
Marja Timmermans, University of Tübingen
Verena Tomasini, Carnegie Institution for Science
Alexandria Tran, University of Illinois
Tuan Tran, Nanyang Technological University
Masamichi Ueda, Graduate University for Advanced Studies
Richard Uhrig, University of Alberta
Gokul Upadhyayula, University of California, Berkeley
Atique Ur Rehman, Bahauddin Zakariya University Multan
Kannan V., Kalasalingam Academy of Research and Education
Papa Rao Vaikuntapu, ICAR - Directorate of Groundnut Research
Frank Van Breusegem, VIB-Ghent University
Sarah Van Dierdonck, Duke University
Tamas Varga, EMSL, Pacific Northwest National Laboratory
Anne Vatén, University of Helsinki
Dusan Velickovic, Pacific Northwest National Laboratory
Tamara Vellosillo, Stanford University
Willian Viana, Stanford University
Josep Vilarrasa Blasi, Stanford University
A.T. Vivek, National Institute of Plant Genome Research
Sriema Walawage, University of California, Davis
Virginia Walbot, Stanford University
Mengying Wang, North Carolina State University
Xiaohong Wang, Agricultural Research Service, United States Department of Agriculture
Yingying Wang, Westlake University
Doreen Ware, Cold Spring Harbor Laboratory
Eric Warme, University of Minnesota, Twin Cities
Erin Weber, University of Wisconsin, Madison
Clifford Weil, National Science Foundation
Matthew Wengler, University of Saskatchewan
James Whelan, La Trobe University
Asela Wijeratne, Arkansas State University
H-S Phillip Wong, Stanford University

Rafal Woycicki, Applied Omics Woycicki
Clay Wright, Virginia Tech
Cheng-Chiang Wu, Harvard University
Tingting Xiang, University of North Carolina, Charlotte
Tingting Xiao, King Abdullah University of Science and Technology
Shouling Xu, Carnegie Institution for Science
Xiaosa Xu, Cold Spring Harbor Laboratory
Ramin Yadegari, University of Arizona
Masashi Yamada, Biotechnology Center in Southern Taiwan, Academia Sinica
Haidong Yan, Virginia Tech
Athena Yi-Chun Yeh, Cornell University
Elaine Yeung, University of California, Riverside
Chan Yul Yoo, University of California, Riverside
Eduardo Zelada, Universidad Peruana Cayetano Heredia
Junpeng Zhan, Donald Danforth Plant Science Center
Xixi Zhang, Institute of Science and Technology Austria
Huan Zhang, University of California, Berkeley
Jing Zhang, University of Helsinki
Lin Zhang, University of California, Davis
Shuxiao Zhang, University of California, Davis
Shiqi Zhang, Boyce Thompson Institute
Chengsong Zhao, North Carolina State University
Feng Zhao, RDP Institute
Haijun Zhao, Noble Research Institute
Qiong Zhao, Chinese University of Hong Kong
Po-Xing Zheng, Agricultural Biotechnology Research Center
Mowei Zhou, Pacific Northwest National Laboratory
Mingxi Zhou, University of Florida
Ying Zhu, Pacific Northwest National Lab
Xiaohong Zhuang, Chinese University of Hong Kong
Gaurav Zinta, Shanghai Center for Plant Stress Biology
Deevita

Appendix 3: Workshop Feedback

Poll Responses During Workshop

Session 1

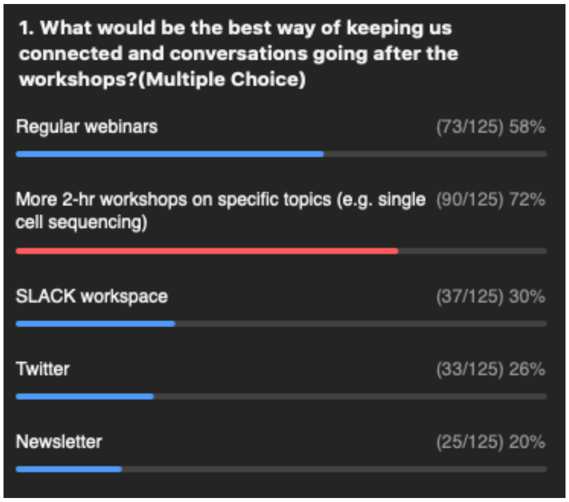

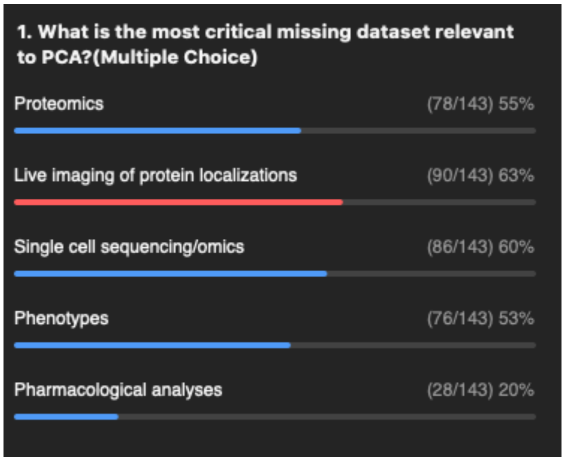

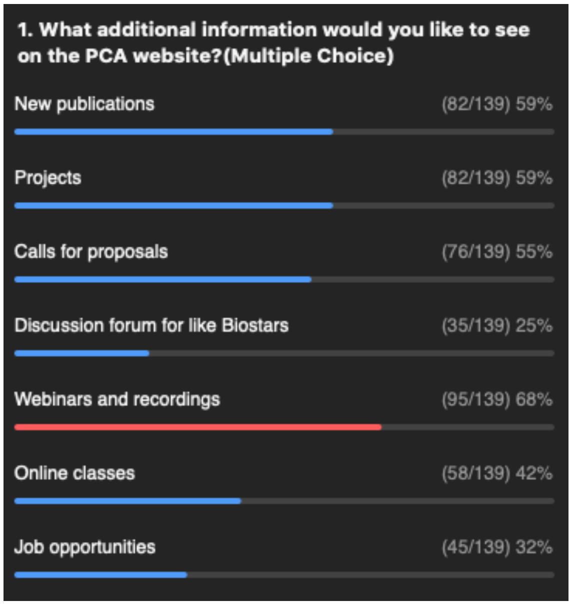

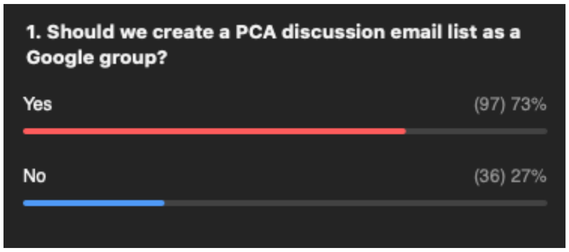

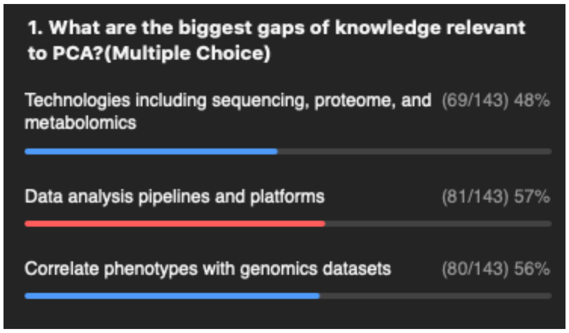

Session 2

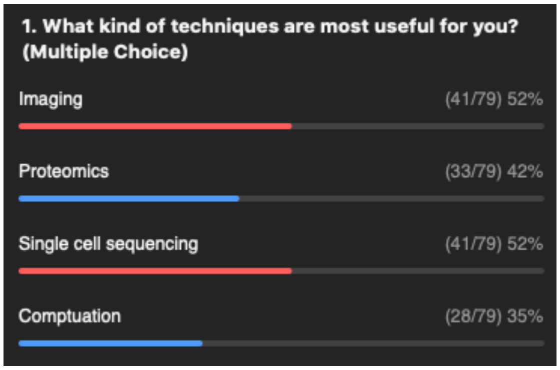
1. What kind of techniques are most useful for you? (Multiple Choice)
Imaging (41/79) 52%
Proteomics (33/79) 42%
Single cell sequencing (41/79) 52%
Comptuation (28/79) 35%

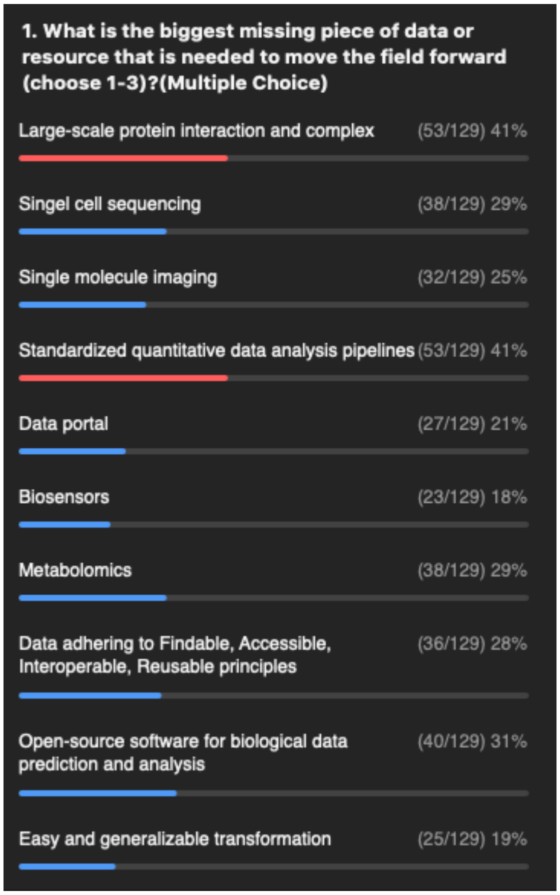
1. What is the biggest missing piece of data or resource that is needed to move the field forward (choose 1-3)?(Multiple Choice)
Large-scale protein interaction and complex (53/129) 41%
Singel cell sequencing (38/129) 29%
Single molecule imaging (32/129) 25%
Standardized quantitative data analysis pipelines (53/129) 41%
Data portal (27/129) 21%
Biosensors (23/129) 18%
Metabolomics (38/129) 29%
Data adhering to Findable, Accessible, Interoperable, Reusable principles (36/129) 28%
Open-source software for biological data prediction and analysis (40/129) 31%
Easy and generalizable transformation (25/129) 19%

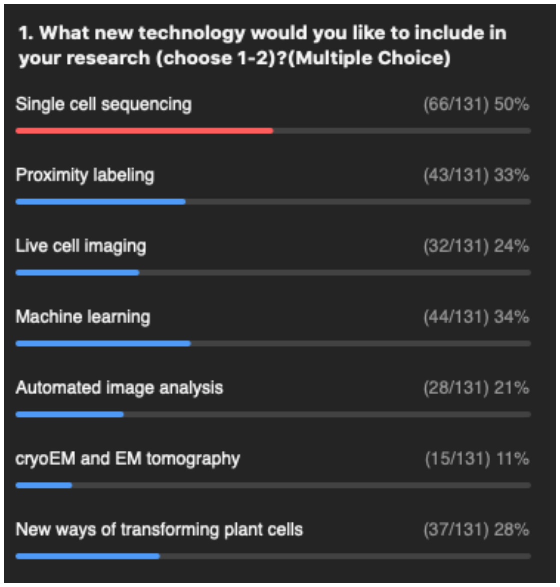
1. What new technology would you like to include in your research (choose 1-2)?(Multiple Choice)
Single cell sequencing (66/131) 50%
Proximity labeling (43/131) 33%
Live cell imaging (32/131) 24%
Machine learning (44/131) 34%
Automated image analysis (28/131) 21%
cryoEM and EM tomography (15/131) 11%
New ways of transforming plant cells (37/131) 28%

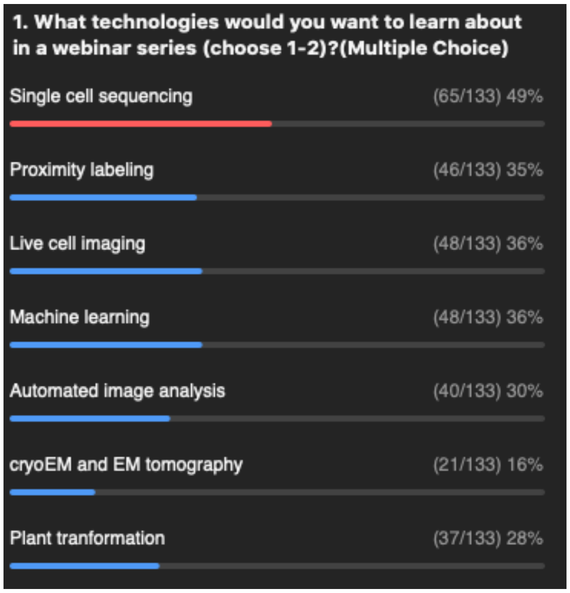
1. What technologies would you want to learn about in a webinar series (choose 1-2)?(Multiple Choice)
Single cell sequencing (65/133) 49%
Proximity labeling (46/133) 35%
Live cell imaging (48/133) 36%
Machine learning (48/133) 36%
Automated image analysis (40/133) 30%
cryoEM and EM tomography (21/133) 16%
Plant tranformation (37/133) 28%

Session 3

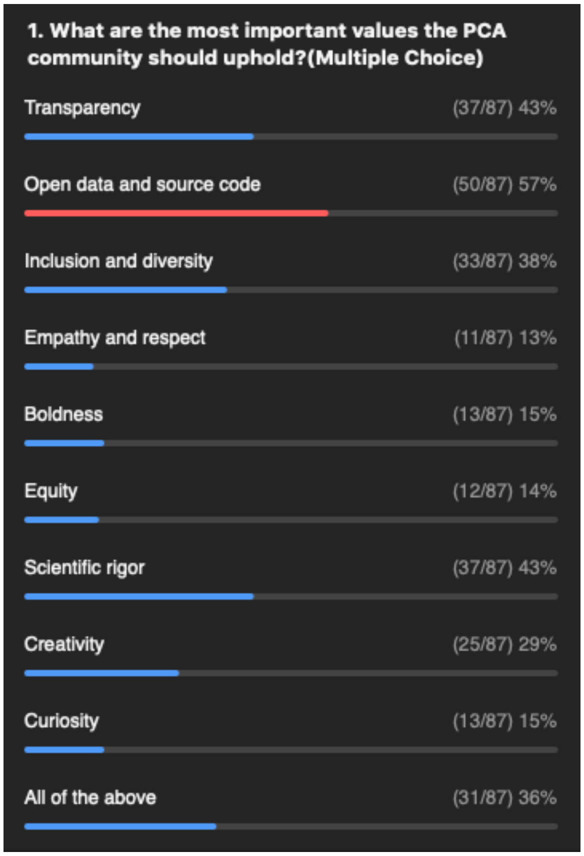
1. What are the most important values the PCA community should uphold?(Multiple Choice)
Transparency (37/87) 43%
Open data and source code (50/87) 57%
Inclusion and diversity (33/87) 38%
Empathy and respect (11/87) 13%
Boldness (13/87) 15%
Equity (12/87) 14%
Scientific rigor (37/87) 43%
Creativity (25/87) 29%
Curiosity (13/87) 15%
All of the above (31/87) 36%

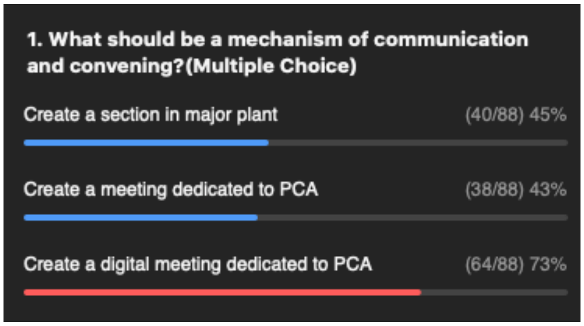
1. What should be a mechanism of communication and convening?(Multiple Choice)
Create a section in major plant (40/88) 45%
Create a meeting dedicated to PCA (38/88) 43%
Create a digital meeting dedicated to PCA (64/88) 73%

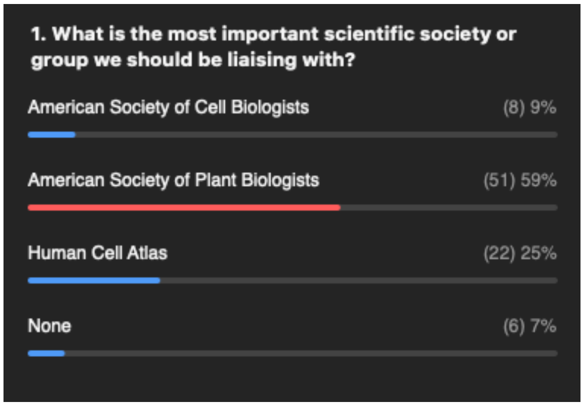
1. What is the most important scientific society or group we should be liaising with?
American Society of Cell Biologists (8) 9%
American Society of Plant Biologists (51) 59%
Human Cell Atlas (22) 25%
None (6) 7%

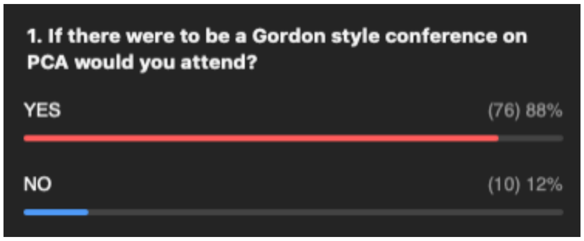
1. If there were to be a Gordon style conference on PCA would you attend?
YES (76) 88%
NO (10) 12%

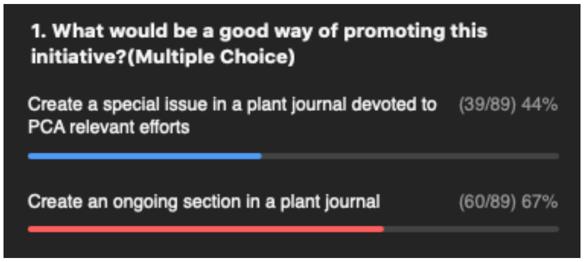
1. What would be a good way of promoting this initiative?(Multiple Choice)
Create a special issue in a plant journal devoted to PCA relevant efforts (39/89) 44%
Create an ongoing section in a plant journal (60/89) 67%

Post-Workshop Survey Responses

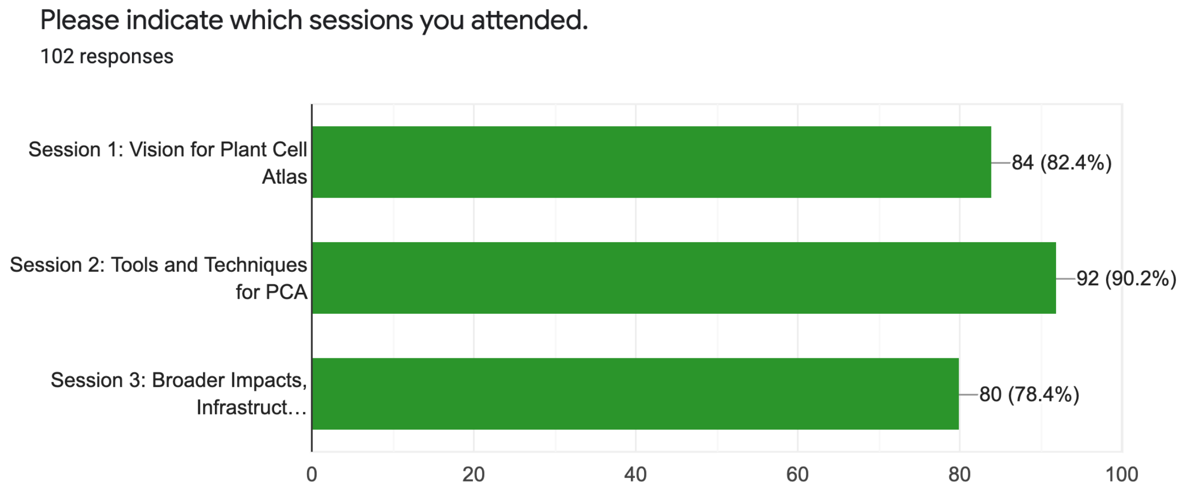

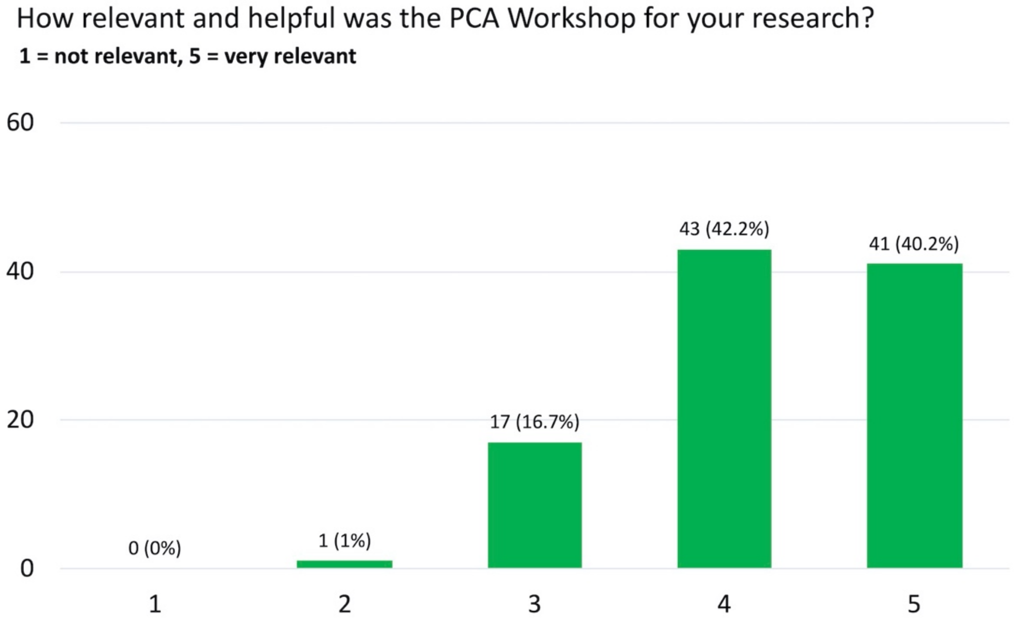

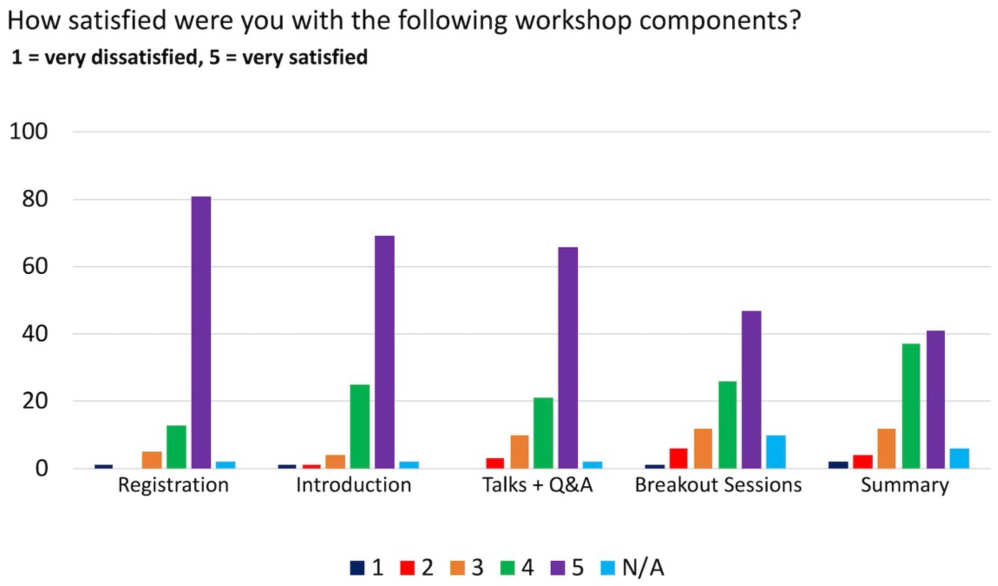

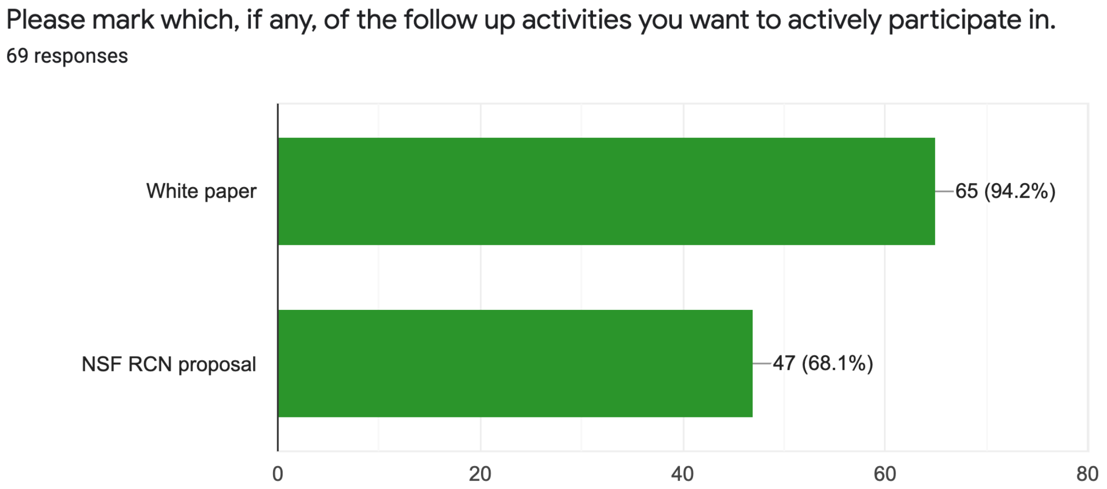

How can we improve the workshop in the future?

- Perhaps break up groups based on experience. My groups were often graduate students that did not participate actively in the conversation.
- Let sessions know the time; I felt rushed; many people had poor connections
- I think the summary sessions have to be improved or changed, because the information holds because of the lack of time. Perhaps it's better to resume and share later the main points of the sessions.
- Too focused on transcript-based measurements. Look beyond Cell Atlas and Allen Brain Atlas for ideas on how to move forward. Look into NIH's HubMAP, KPMP, and LungMAP.
- There were some technical difficulties but it was nice that everything was online.
- More explanation of how invited talks illustrate the workshop theme.
- As the concept of PCA becomes defined it might be better to have talks focused more on that.
- Think more in the time of the event.

- A little bit longer for the talks. The research is very interesting, but most folks could only superficially introduce their work before time was up.
- External panel or speaker from non-basic research stakeholders: industry, producer, breeder from different agriculture sectors.
- There were no networking opportunities which was really disappointing for graduate students who are looking for jobs (postdocs).
- It may be fine to increase the number of talks (1-2 more).
- Longer expert lectures, more time to summarize the discussion sessions - but I think the time we had was used very effectively.
- Please try to share the research articles published for better understanding.
- I would like to see longer talks that allow us to get more details of the current technologies, types of data, and methods, etc.
- Introducing topics that are more to the level of early researchers or those just coming into the field. At some point, I got lost on some of the things shared.
- Thanks so much for putting together a very informative virtual workshop.
- More focused sessions, with less, but longer presentations. Maybe inserting a small presentation after the breakout sessions to help people summarize the discussions and have a better summary part.
- Technical workshops that can introduce and teach new techniques will be great. :)
- The talks are too short. I think the talks should be around 15 minutes.
- I would have wished to hear the vision for single cell application in each of the talks. This would have given more insights into the goals and concept of the PCA. If possible, it would be nice to organize break-out rooms in the 30 min before the meeting starts that allow early career researchers to connect with experts in the field (e.g. the presenters of the talks from the previous session).
- I thought things were quite good, except the summaries were a bit discombobulating with the live documents from the moderator/scribe perspective. Not sure of how to do this in a better way. Perhaps if there were some other activity to most participants, while moderators and scribed summarized the breakout prior to reporting?
- I think at the last session we nailed with an appropriate number of participants per room.
- The summary after the breakout sessions was not really useful, maybe a better way to wrap up.
- I think having instructions for breakout moderators on how to ensure that everyone has a chance to speak would be helpful. I thought it was GREAT having so many small breakout groups so should be easier to ensure people have shared. Also the live notes were kind of a pain because all the editing makes the page jump when you are writing (I was a scribe.)
- If it is going to remain virtual, look into innovative features that could only be done virtually (the polling was a good example of this). Also a poster session could be cool.
- Breakout sessions could be optimized; groups could be organized around a research theme or question. Additionally, the number and breadth of questions for discussion was too much for the time allotted. Need more time, fewer questions, or more specific questions (or a combination).
- So many things like having clear objectives and don't read Powerpoint and notes.
- I wonder if there's a better way to handle breakout rooms in the future. There has been an issue in every session that I've attended.
- For recent undergraduate students like me, it would be helpful to introduce common technologies used for building a PCA.
- I liked the format and thought it worked well.
- The breakout sessions and summary were great and improved during the workshop. However, there can be a room to improve. If we can have a follow-up information after the meeting including the Google slides documents etc. might be great.

- More please.
- For the first workshop in which an overview was provided, it was great. Future workshops should probably have fewer, but slightly longer talks so more details can be learned about particular techniques.
- Have a better vision to communicate about the purpose of PCA.
- Maybe engage more people developing new technology (like inviting researchers to speak). This would foster more interdisciplinary collaborations.
- If possible to extend the time allotted to each session, having somewhat longer talks and/or more presenters might have been good.
- In a form of more specific subtopics.
- More segmentation of different topics besides the 3 in the 3 workshops.
- I think it would help to find researchers capable of bridging various types of datasets. This would help scientists think more about data integration.
- More time for reflection, or summary/integration of the discussion outputs.
- I think it was really well done and would recommend using the same format for future workshops.
- Maybe have longer versions of the talks pre-recorded (together with the shorter ones live).
- Reach out as much in social media.
- Some of the breakout session questions seemed similar, so more variety/specific questions. I thought the summary should happen in a different session with put together slides /a guided conversation.
- A suggestion: A section may be included for each participant to write about their areas of expertise under broadheads.
- I feel it should be more focused.
- More substantial
- It might be worth spending a little bit more time on the summary at the end of the workshop. It is very interesting to see what other people have discussed, so covering more different groups might be good.
- Can we have a more specific research field-focused workshop?
- Focus effort and resources on a few specific plants/technologies rather than encompassing multiple sub-disciplines, technologies, and species. Or split the PCA into a few sub-groups with specific aims and interests for each.
- Talks were too short to grasp the important points.
- The event was very successful, though I would have set a better framework. I would have used an introductory one slide similar for all talks, to make comfortable participants from various scientific backgrounds, but not familiar with plant biology.
- Having a session of open discussion would be very nice. I know it can be complicated with many participants on Zoom, but it should be doable if attendees do not speak on top of each other.
- Share meeting minutes and action points after each session.
- Consider including discussion on options for translation of PCA insights to societal benefits at each workshop.
- Consider building a catalog of participant profiles to promote interactions amongst participants, also beyond the workshop sessions. Ideally, this could be extended to developing a communication and ideation platform at which people can submit and discuss ideas. MS Teams could be an easy way to go. An even better option would be to consider a dedicated ideation application (Hype, Yambla, ...).
- It was well conducted.
- I think the breakout sessions could have been a little longer, as there was a lot to discuss. In addition, it would have been nice to have a break between the breakout session and the summary for scribes to finish writing summaries.

- Longer research talks.
- Increase the talk time, 20 minutes for each speaker is short.
- It would be nice if attendees could get the summary of discussion.
- You can also include a topic of the role of plant cells considering it on the behalf of environmental science. .
- The workshop was very well organized. I can't think of anything.
- Minimize breakout session.
- Please increase the talk and Q&A session.

Any overall feedback for the event (what worked well and what didn't work well)?

- The use of Zoom worked well and allowed excellent participation.
- Best possible outcome given the circumstances. Well organized. Well executed. Well done.
- Great work given the circumstances of COVID19.
- Breakout sessions were the weakest part.
- There were some issues with breakout groups (not all folks stayed on, not everyone could be heard due to technical difficulties), but overall the workshop was interesting and worth my time.
- I think the talks were very good ones!
- The rapid and balanced assignment of individuals to working groups (also note that many individuals were part of the Zoom call but did not participate). In that sense, groups could have been larger.
- Look forward to it being in person next time. Otherwise it went well.
- The rapid and balanced assignment of individuals to working groups (also note that many individuals were part of the zoom call but did not participate). In that sense, groups could have been larger.
- I thought the breakout room workshops worked well. The speaker sessions were a bit short though.
- The breakout assignments worked well in random mode and failed when attempts were made to assign specific people.
- I loved to hear the 5-minute conferences. They were long enough for getting to the point, but short enough to still have the public's attention.
- Zoom breakout rooms were great! Gave a very large meeting a very personal feel.
- Very well-organized event.
- Great talks but they seem rushed (too short). No networking which is the best part of attending a conference.
- Breakout sessions were particularly wonderful. It could be better to keep the same group across sessions so that each member can get to know each other well in the next sessions and participate in the discussions proactively. Some people barely participated in the conversation.
- it was a great opportunity for colleagues and exchange ideas - it would be wonderful to get to meet in person sometime soon.
- I was very satisfied with the workshop event, very recent and advanced research was presented. I have great hopes to learn much in future.
- Questions that come to my mind (as a grad student) is, how I can contribute to the project? How can I get involved? Are there positions available related to the project? Could it be part of a publication? With that said, I think that at some point it would be nice to have events (iGEM-like) to bring the whole community into specific problems/questions related to PCA.
- Better communication. But it was enlightening as well. That is for sure.
- It would be nice to have a recorded session and provide the recording upon request for someone (like me) who could not attend other sessions.

- The break-out sessions were great but hard to get started the first two workshops.
- I really liked the breakout sessions and the questionnaires afterwards, however the summary part was a bit confusing, as people were continuously writing to the files and it was difficult to follow.
- Pretty much liked everything. No complaints. :)
- I think the workshop should encourage more undergrad and graduate students to participate.
- I think the 5 minute talks were perfect.
- It was enough time to tell a story, and not too much time for losing the attention of the public.
- Seven-minute or even ten-minute talks could also work.
- I very much enjoyed the short talks and the comparatively long break-out sessions. Also technically, this was organized very well.
- Participation was low at the breakout sessions that I was assigned. They had 5-6 people and only 2-3 were involved in active discussions. Maybe next time, there could be less sessions with more participants.
- I think the short talks, break out sessions, and then return, worked really well.
- Maybe a small pause between talks to make sure people can write down their questions.
- I think breakout chat groups worked really well (apart from hickups in assigning scribes and moderators).
- The virtual format worked great. The mix of talks and breakout sessions was a good balance. Even though the technologies weren’t perfect I still thought it all worked out.
- The discussions in the breakout sessions were the best part. Good priming by the short talks at the beginning.
- I thought it went great; more so considering that it wasn't originally planned to be fully virtual.
- I thought it went very well and really appreciated seeing how you adjusted each workshop (wrt breakout rooms and opting in or out of participation in those.) As noted the note taking was a bit janky so the way folks modified was to copy over the notes into a document and then paste back into the mail document. The summary slides were kind of problematic for me because at the time I wanted to be listening to what ppl were saying so I had to transcribe a summary. The template could include a summary section that could also be pasted in.
- The lineup was fantastic. Going over time and technical difficulties were problems. If recordings were easier to access that would also be great.
- Great to discuss big ideas with people I didn't know before, and a great opportunity for inclusion of ECRs and those from smaller institutions.
- Breakout rooms on day 3 were the most organized.
- All work is good.
- The 5-minute talks were really great.
- Please give us a restroom break in the middle. It's difficult when we don't want to miss anything.
- The feedback with the slides, and feedback, and polling, was a bit overwhelming.
- I enjoyed the high quality talks and the discussion during the breakout sessions. It was impressive to see the organization is kept improved in each session.
- Fantastic, really appreciate it!!
- Organizers did a great job given that the workshop was virtual. breakouts were great. Not much to say about what did not work.
- I was initially skeptical how the short introduction talks would work because 10 min is really not that much. But I was impressed that most speakers did an amazing job to give a thorough introduction into the topic! Also, I thought the organization through Zoom with the plenary introductions and the breakout rooms was working really well! Only, for the

scribes, I found the shared Google document difficult to work with. Everybody was writing in the same document, the paragraph for each group was constantly shifting and moving.

- The breakout session was a nice idea and fun, but seemed geared toward finding an identity for the PCA.
- The breakout session was very helpful.
- The remote Zoom session was handled very well, use of chat for collecting questions and breakout rooms. Nice to see the postdocs taking responsibility for various components, they did a very good job of keeping things on track.
- it went really well! I really enjoyed it! Thanks so much!
- The access to the breakout sessions during the second session was really difficult
- I think it worked very well - I am looking forward to hearing about the summary of discussion outputs.
- All of it really did work well. Very professional and there was rapid recovery from slight snafus.
- Very good.
- An excellent workshop to get scientists on the same platform to initiate PCA.
- Too broad
- Maybe too broad, but is really a good start for an integrated level.
- Overall worked well.
- The talks at the beginning of each workshop were excellent and the chat function worked well for questions. More specific questions would have been helpful to facilitate discussion in the breakout sessions.
- Overall very successful!
- The event was very successful even considering its webinar version -not easy- re-organization. Great job! Brief talks and Breakout Rooms were really good ideas. I would have asked for a more strict home assignment before a new session.
- Everything worked well in my opinion.
- Introductory presentations by domain experts is an excellent starting point for breakout sessions.
- Experts displayed a high level of enthusiasm which is highly motivating and encouraging to early career scientists.
- The virtual talks/breakout sessions worked surprisingly well!
- Breakout sessions were overall good, but depended on the activity of the participants.
- It will be better if you can provide the link for recording of every talk.
- Thank you for organizing such an excellent workshop. We discussed it related to a bit but I would like to know what the vision of the PCA committee or organizers is.
- I enjoyed the interaction possibilities, meeting people and listening to the talks providing various different perspectives.
- Excellent
- The format was well thought out and implemented. I enjoyed the short talks setting the context for break-out room discussions. It was very intellectually stimulating to hear the speakers talking about their visions of PCA and its significance for their areas.
- Breakout sessions were good.

What are the types of technologies you would like to hear about in a webinar?

- Single-cell sequencing.
- Single cell Epigenomics
- Comparison of cell preparation methods; comparative costs for all steps
- Consider inviting non-plant folks to talk about the latest technologies that have not yet made it to plants to encourage folks to take on the challenge of adapting them to plants!

- New approaches to do a standardized transcriptome (or proteome...) atlas and dismiss the errors in the past, for example: bad annotations in databases, bias in the transcriptomes. And also new algorithms to deal with transcriptomics data and modelling.
- High-resolution microscopy, Single cell -omics, Topics establishing the relationship between genotype and phenotype, large scale phenotyping.
- Metabolomics.
- ML & graph databases.
- Stuff that is useful for maize research.
- Spatial transcriptomics; Tissue-scale live cell imaging and modeling
- metabolomics, proteomics, imaging.
- Computational modeling and data integration.
- advanced imaging, plant-centered proteomics, metabolomics.
- Live Imaging and advanced instrumentation.
- scRNA-seq, scATAC-seq and scChIP-seq (it is available).
- More on computational analyses; more on single cell technologies.
- Single cell sequencing (RNA, ATAC, multimodal, etc.). Imaging techniques (e.g live imaging vs clearing/staining). Computational modeling, segmentation.
- Single-cell sequencing.
- Proteomics.
- Molecular biology or phytochemistry techniques.
- protocols and benchmarking for scRNA-Seq and snRNA-Seq.
- Single-cell metabolomics.
- PCR overview.
- scRNA-seq and other single-cell methods.
- Image analysis, machine learning.
- ATAC-seq, more on Turbo-ID, more on live imaging.
- Data integration; Single cell omics; Quantitative imaging.
- More than just single cell sequencing - tools and techniques for studying individual cell types - like TurboID or INTACT, for example.
- So many like metabolism, CRISPR, proteomics,MALDI-TOF, single cell sequencing, TRAP, real time imaging etc.
- Advances in imaging technology.
- Interactive technologies like flash.
- Technologies to analyze special organization of metabolites and metabolic pathways in subcellular, cellular, tissue, and organism levels.
- Single-cell seq.
- Epigenome editing
- Protein-protein interactions, single cell omics, strategies for translating plant cell biology research to agricultural outcomes, new live cell imaging modules.
- proteomics, micro-proteomics.
- Integration of scOMICS data, analytical methods, DBs, resources, core labs
- Mass spectrometry, cryoEM
- Spatially resolved transcriptomics; integrative analytical approaches.
- The method of building networks.
- DNA, gene regulation, how to assign cell types, what are the regulators of cell types
- Computational tools (e.g., Seurat, Monocle 3).
- Molecular imaging techniques.
- Subcellular 3D tomography, with protein localization (I guess with super resolution microscopy?).
- Faster and more efficient transformation or other approaches for functional testing of genes.

- It would be interesting to hear from state-of-the-art projects outside of plants.
- Advancements in super resolution microscopy in plants cell biology.
- Hard to pick: modeling and bioinformatics tools, sequencing techniques, methods for chemical analysis.
- Emerging technologies.
- scRNAseq, single-cell anything.
- (cryo-)electron tomography.
- Single-cell sequencing, microscopy.
- Single-cell expression analysis.
- scRNA-seq experiments and data analysis.
- Cryo-electron tomography is a technique where an electron microscope is used to record a series of two-dimensional images as a biological sample held at cryogenic temperatures is tilted.
- This technique has the advantage to determine the structure of cellular machinery inside the cell avoiding the damage to the complexes during purification. This technique aims to provide information on cross-talk of the complexes with their cellular components.
- Plant optogenetics.
- scRNA-seq and spatial transcriptomics approaches (at any level) in plants.
- Biosensors.
- Technologies to map protein interactions and complexes.
- Technologies to visualize gene and protein expression (regulation).
- Methods for non-destructive analysis of cellular processes (at the single-cell level)
- transcriptomics, epigenetics, evo-devo, cell and developmental biology.
- More spatial transcriptomics/metabolomics methods (e.g., Spatial Transcriptomics, or MALDI imaging).
- scATAC-seq.
- Single-cell sequencing, bioinformatics.
- cryo-TM.
- Spatial modelling, single cell and spatial genomics, high resolution microscopy
- (as well as their applications to biological questions in plants).
- Related to plant cell *i.e.* DNA Marker, various production technologies and tools etc
- Spatial transcriptomics, gene editing, computational modeling at molecular, cellular and organismal scales.
- Single cellomics, advancements in Imaging technologies, data analysis.
- Genome editing.

What is your dream for the PCA?

- That it will generate excitement for the topic that will propagate through future conferences (Arabidopsis, maize *etc.*) as a theme in workshops etc.
- The PCA must have the ability to grow and incorporate information from contributions of labs ranging in size and ability. There are too many cell types, growth stages, and growth conditions to do it all at once. So, my suggestion is this - for any technology or experiment type, we should set the standard for how the data should be handled, curated, processed, etc. and make the pipelines re-usable. This way, any other lab can perform their experiment, on their plant, in their conditions, on their cell type, and simply drag-and-drop their dataset into the pipeline, and into the PCA it goes. And this way you know all the datasets are comparable.
- Accessible, normalized datasets for meta-analyses and deriving new solutions.
- Resource for sharing protocols, tech tips, data.
- That we have it fully populated and can do "research" during the global shutdown by just clicking on PCA icons without touching a live plant ;-).

- PCA gives us the possibility of creating a very powerful tool, not only for the access and/or visualization of the information that is provided in the PCA, also for using this information in new pipelines and algorithms that let us to generate new knowledge. I would focus in some items, and here I highlight some of my ideals:
  1- Create a cellular atlas combining the information from multiple studies and use algorithms for the normalization and extraction of the characteristics of the cell type.
  2- Maintain the original datasets with all the growth conditions, and development stage, very well annotated, creating a standard form that led us to use this information without the need of a manual annotation.
  3- Expand PCA to multiple species
  4- Consensus in data format
- That it becomes a milestone platform in plant biology like TAIR has been.
- My dream for PCA - for it to be a comprehensive platform of integrated data from different plant species, which will be instrumental in translating basic plant science research to bring about advancements in agriculture, medicine, and bioenergy. Additionally, I hope to contribute to building PCA as a modular and dynamic resource that will be a primary driving force in bringing about policy changes and how plant science research is funded in the future.
- To be consortia of many plant cell atlas efforts, each funded by different agencies.
- That becomes a milestone platform in plant biology like TAIR has been.
- Unified exemplar.
- I would like the PCA to be an alternative to the literature as well as a gateway to the literature. I would like to query a gene and be able to drill through high-quality data for all three ontological categories. This should include the organ and subcellular locations of its products; molecular interactors; post-translational modifications; OE and KO phenotypes; roles and functions.
- For the PCA to become an integrative research community that establishes standards, collates datasets/models, and celebrates achievements that work towards a complete positional and temporal understanding of plant growth and development.
- Cell expression atlas for most major plant species.
- To have an online resource to find the location of proteins and metabolites in plant cells, linked to their functions.
- Engagement in the community and contribution to the goal.
- An integrative understanding of plant function, from molecules to an entire organism, to understand how different cell types are generated and give rise to diversity.
- I believe like HCA, PCA would allow better and high-resolution understanding of deep mechanisms in plant cell functions which could allow for the crop engineering.
- Track the regulatory events that define the cell trajectory from seed cells until the establishment mature plants.
- To be part of the researchers that bring about a change to the world of plant science and technologies. I believe the field is vast and has a lot of potentials that are yet to be explored. I want to be part of that transition and awareness to other students.
- 1, To understand plant cell types and their interaction with each other to make tissues, organs, different shapes and functions.
  2, To establish and promote new communities and collaborations to bring cell level plant research forward.
  3, In a more technical note, to establish and promote standardized workflows for single cell level analysis in plants, which could be applied to a wide range of species in almost any laboratories.
- Establish a shared database for all the omics data.
- That we can generate a data-rich resource base that provides information on transcriptome, epigenetic, proteome and metabolome features of cells /cell-types.

- Incentive and infrastructure for collecting data and building an integrated model of plant growth development and evolution.
- To assemble both spatial and temporal data into a unified framework.
- An integrated digital environment containing multi-omic data sets that allows seamless visualization, and modeling at a click!
- Integration of the community across scales.
- A user-friendly resource, accessible to all, that will facilitate and accelerate our research.
- To grow into more than a data resource. I would like to see this as an opportunity to foster training, networking, and collaborations.
- To also be applicable to non-model organisms to facilitate the rapid domestication of plants.
- A resource that enables integration of quality data with value greater than the sum of its parts - enabling multi-scale modeling, etc.
- Collaboration.
- I would love it to be a central resource for plant cell biology, which would aid both research, standardize format of published data, and teaching.
- For students like me, the PCA would be extremely useful for tracking what we know about plant cells. A lot of the materials I know are textbook material. It would be great if there was a virtual guide from the eye to cell level of what we know of plants today.
- To have a body of cell specific, and subcellular specific, information of protein function and its effect on cellular processes. Further, to have a framework (data repository, analysis pipelines, standard protocols) through which data sets can be collated and compared.
- 1. Determining concentrations of metabolites in all subcellular locations in all cell types in plants.
  2. Precise and reliable protein-protein interaction map for the metabolic enzyme complexes.
- New tool that empowers us all as a community so we can accelerate research in time for climate change in plant sciences.
- A comprehensive resource (tools, training *etc.*) for current and early career plant scientists.
- That it's really easy and accessible. The eFP browser does a fantastic job here for their data. The easier the PCA is to work with, the more it will be used and the likelier it is that it will also be a useful tool for teaching.
- Translation of cell specific data to trait crop trait level knowledge.
- A virtual environment with the ability to represent developmental trajectories starting from fertilization and allowing overlay of information from different 'omics approaches, as well as dynamic responses to environmental factors, across a wide variety of species (and eventually multiple genetic backgrounds within species). The final aim would be to integrate as much as possible about the knowledge of cell structures
- To create a website integrating our knowledge of plant single cells.
- Cellular structures mapped out, with differential forms captured for different cell types and environmental conditions - and their dynamics! Extra plus if organellar proteomes are identified...
- Centralization of tools and knowledge for a wide range of plant species with a user friendly, searchable interface (keep talking to Provart!).
- Communal database to which people upload information and tips too. Would love to see a null results database (share what didn't work, alongside what does) and a list of software/resources people use for various things (for example, automating things and data processing/pipelines). It would also be nice to share meta data/have more information for reproducing results and to standardize testing/extracting/processing procedures and terminology in the field.

- A one-stop user-friendly website for:
  a) data on genes and their networks in multiple plants, and their organs/tissues
  b) tools for big data and image analyses
- Discovering new cool biology.
- Maybe integrated encyclopedia.
- For it to be a community resource that inspires new research questions, approaches, and collaborations.
- PCA is the first thing that comes into people's heads when they think about a plant cell.
- A streamlined database that houses and organizes single cell data. A vibrant community that facilitates interaction between bench and computational scientists.
- Connecting research tools to new users.
- PCA integrates molecular biology, biochemistry and bioinformatics within an ontological framework to produce a reference database that is both functional and practical for plant researchers, agricultural agencies, food industries and other related industrial applications.
- PCA project seeks a collaborative international network effort to indicate the areas of impacts of plant biology and agriculture. PCA should provide the education and training materials for researchers and public to inspire future generations.
- PCA should provide an ontology framework consisting of a precise vocabulary for the descriptions of plant cell compartments and signaling to allow researchers cross-talk in need to share a variety of available data.
- PCA should seek the collaboration of scientific illustrators as prof. David Goodsell is an associate professor at Scripps who has rendered many molecular machines with a graceful recognizable style. He has written public molecular graphics programs to visualize experimental protein, DNA and complex structures of these macromolecules.
- PCA mission should include innovative aspects of the work of many from different geographic areas dealing with crop domestication and agriculture.
- It would be great if the PCA will work in light of the United Nations Sustainable Development Goals.
- PCA = a community of open-minded plant aficionados building a cell-level understanding of processes defining the plant phenotype as the basis for sustainable food production.
- become a hub for education.
- A community-organized virtual center that funds, curates, hosts, and standardizes data from high-resolution emerging methods.
- A consortium-like group for method and data standardization and curation
- Create a network of expert scientists to establish a comprehensive, accessible resource mapping cellular gene activities profiles across model and crop plant species
- To enable precise and predictive engineering of plant genomes to improve agricultural productivity and resilience

Appendix 4: The Plant Cell Atlas Consortium

Name	Affiliation	Email
Seung Yon (Sue) Rhee	Carnegie Institution for Science, CA, USA	srhee@carnegiescience.edu
Selena Rice	Carnegie Institution for Science, CA, USA	srice@carnegiescience.edu
Suryatapa Ghosh Jha	Carnegie Institution for Science, CA, USA	sjha@carnegiescience.edu
Anna Stepanova	North Carolina State University	atstepan@ncsu.edu
Sanjay Joshi	University of Kentucky	sjo333@g.uky.edu
Aaron Ogden	PNNL	Aaron.Ogden@pnnl.gov
Jennifer Brophy	Stanford University	jbrophy@stanford.edu
Batthula Vijaya Lakshmi Vadde	Cornell University	vb336@cornell.edu
Tatsuya Nobori	Salk Institute	tnobori@salk.edu
Andrey Malkovskiy	Carnegie Institution for Science, CA, USA	amalkovskiy@carnegiescience.edu
Tie Liu	University of Florida	tieliu@ufl.edu
Pinky Agarwal	National Institute of Plant Genome Research, India	pinky.agarwal@nipgr.ac.in
Mather A Khan	University of Missouri	khanma@missouri.edu
Shyam Solanki	Washington State University, Pullman, WA	shyam.solanki@wsu.edu
Pradeep Kumar	CSIR-CCMB, Hyderabad, India	pdpk123@gmail.com
Dominique Bergmann	Stanford University/HHMI	dbergmann@stanford.edu
Gergo Palfalvi	National Institute for Basic Biology, Japan	palfalvi@nibb.ac.jp
Atique ur Rehman Pankaj Kumar	Bahauddin Zakariya university CSIR-IHBT, Palampur<br>Himachal Pradesh India	dr.atique@bzu.edu.pk<br>pksharmabiotech@gmail.com
Jahed Ahmed	Louvain Institute of Biomolecular Science and Technology, UCLouvain, Croix du Sud 4-L7.07.14, B-1348 Louvain-la-Neuve, Belgium.	jahed.ahmed@uclouvain.be
Elsa Herminia Quezada Rodríguez	Universidad Nacional Autónoma de México	qrelsa@gmail.com<br>qrelsa@comunidad.unam.mx
Papa Rao Vaikuntapu	Indian Council of Agricultural Research (ICAR-DGR), Gujarat, India	paparaovaikuntapu@gmail.com
Trevor Nolan	Duke University	trevor.nolan@duke.edu
Maria Harrison	Boyce Thompson Institute	mjh78@cornell.edu
Dae Kwan Ko	Michigan State University	dkko@msu.edu
Christopher R. Anderton	Pacific Northwest National Laboratory	christopher.anderton@pnnl.gov

Javier Brumos	North Carolina State University	jbrumos@ncsu.edu
Gozde Demirer	University of California, Davis	gsultandemirer@gmail.com
Kaushal Kumar Bhati	Louvain Institute of Biomolecular Science and Technology, UCLouvain, Croix du Sud 4-L7.07.14, Louvain-la-Neuve, Belgium	kaushal.bhati@uclouvain.be
Elaine Yeung	University of California, Riverside	eyeun001@ucr.edu
Rachel Shahan	Duke University	rms73@duke.edu
Gazala Ameen	Washington State University, Pullman, WA	gazala.ameen@wsu.edu
Alexander T. Borowsky	University of California, Riverside	aboro002@ucr.edu
Mowei Zhou	Pacific Northwest National Laboratory	mowei.zhou@pnnl.gov
Kangmei Zhao	Carnegie Institution for Science, CA, USA	kzhao@carnegiescience.edu
Felix Rico-Resendiz	LANGEBIO-Cinvestav	edgardo.rico@cinvestav.mx
Sakil Mahmud	University of Bonn	mahmudsakilbau@gmail.com
Steven Salvini	Edinburgh, Scotland	stevensalvini@gmail.com
Marcela Rojas-Pierce	North Carolina State University	mrojasp@ncsu.edu
Ahmet Bakirbas	University of Massachusetts Amherst	abakirbas@umass.edu
Steven Briggs	University of California, San Diego	sbriggs@ucsd.edu
Devang Mehta	University of Alberta	devangmehta@ualberta.ca
Richard Glen Uhrig	University of Alberta	ruhrig@ualberta.ca
Clay Wright	Virginia Tech	wrightrc@vt.edu
Claire McWhite	The University of Texas at Austin	claire.mcwhite@utexas.edu
Amir H. Ahkami	Pacific Northwest National Laboratory	amir.ahkami@pnnl.gov
Marcela K. Tello-Ruiz	Cold Spring Harbor Laboratory	telloruiz@cshl.edu
Kerstin Kaufmann	Humboldt-Universitaet zu Berlin	kerstin.kaufmann@hu-berlinde
Isil Erbasol Serbes	University of Bremen	isilerbasol@gmail.com
Sergio Alan Cervantes-Pérez	LANGEBIO-CINVESTAV, México	sergio.cervantes@cinvestav.mx
Noah Fahlgren	Donald Danforth Plant Science Center	nfahlgren@danforthcenter.org
Alexander jones	University of Cambridge	alexander.jones@slcu.cam.ac.uk
Tedrick Thomas Salim Lew	Massachusetts Institute of Technology	tedrick@mit.edu
Cesar Cuevas-Velazquez	Facultad de Química, UNAM	cuevas@quimica.unam.mx
Josh Strable	Cornell Univ.	jjs369@cornell.edu
Bastiaan Bargmann	Virginia Tech	bastiaan@vt.edu

Lisa David	University of Florida	lisaidavid@ufl.edu
Nicholas Provart	University of Toronto/Centre for the Analysis of Genome Evolution and Function	nicholas.provart@utoronto.ca
Uwe Ohler	Humboldt University & Max Delbruck Center Berlin	uwe.ohler@mdc-berlin.de
Maite Saura-Sanchez	IFEVA (CONICET - UBA)	saurasanchez@agro.uba.ar
Toshihiro Obata	University of Nebraska-Lincoln	tobata2@unl.edu
Navadeep Boruah	Carnegie Institution for Science, USA	nboruah@carnegiescience.edu
Tamas Varga	EMSL, Pacific Northwest National Laboratory, US	tamas.varga@pnnl.gov
Travis Lee	Salk Institute	trlee@salk.edu
James Whelan	La Trobe University	j.whelan@latrobe.edu.au
Sumedha Arora	Punjab Agricultural University	arorasumedha60@gmail.com
Harmanpreet Kaur	Punjab Agricultural University	hpreet346@gmail.com
Amandeep Kaur	Punjab Agricultural	aman-coasab@pau.edu
Peter Denolf	BASF	peter.denolf@basf.com
Jenny Mortimer	Lawrence Berkeley National Laboratory	jcmortimer@lbl.gov
Dhruv Lavania	University of Alberta	lavania@ualberta.ca
Dianyi Liu	Donald Danforth Plant Science Center & University of Missouri	dliu@danforthcenter.org
Christophe Liseron-Monfils	BASF	christophe.liseron-monfils@basf.com
Javier A Miret	University of Reading	j.miretbarrio@reading.ac.uk
Shipra Goel	Delhi Technological University	shiprag96@gmail.com
Fabio Gomez-Cano	Michigan State University	gomezcan@msu.edu
Purva Karia	University of Toronto	purva.karia@mail.utoronto.ca
Juan Pablo Giraldo	University of California, Riverside	juanpablo.giraldo@ucr.edu
Concepcion Manzano	University of California, Davis	manzano.concepcion@gmail.com

Yongxian Lu	Carnegie Institution of Science	yxlu@carnegiescience.edu
Mingkee Achom	Cornell University	ma2292@cornell.edu
Ramin Yadegari	University of Arizona	yadegari@arizona.edu
Elena Lazarus	Carnegie Institution for Science, CA, USA	elazarus@carnegiescience.edu
Chen Kuang	Chinese Academy of Agricultral sciences	8210181029@cass.cn
Jaishri Rubina Das	National Institute of Plant Genome Research, New Delhi, India	jaishrirubina@nipgr.ac.in
Sofia Otero	University of Cambridge	sofia.otero@slcu.cam.ac.uk
Matthieu Bourdon	University of Cambridge	matthieu.bourdon@slcu.cam.ac.uk
Rajveer Singh	Punjab Agricultural university,Ludhiana, India	rgrewal372@gmail.com
Lothar Kalmbach	University of Cambridge	lothar.kalmbach@slcu.cam.ac.uk
Sagar Kumar	Punjabi University,Patiala, India	sagarsukhija65@gmail.com
Alvaro Daniel Fernandez-Fernandez	VIB-UGent Center for Plant Systems Biology	alfer@psb.ugent.be
Lidor Shaar-Moshe	University of California, Davis	lidorshaar@gmail.com
Renate Weizbauer	Carnegie Institution for Science, CA, USA	rweizbauer@ciw.edu
Chai Hao Chiu	Department of Plant Sciences, University of Cambridge	chc59@cam.ac.uk
Diane E. Dickel	Lawrence Berkeley National Laboratory	dedickel@lbl.gov
